# Conserved host response to highly pathogenic avian influenza virus infection in human cell culture, mouse and macaque model systems

**DOI:** 10.1186/1752-0509-5-190

**Published:** 2011-11-11

**Authors:** Jason E McDermott, Harish Shankaran, Amie J Eisfeld, Sarah E Belisle, Gabriele Neuman, Chengjun Li, Shannon McWeeney, Carol Sabourin, Yoshihiro Kawaoka, Michael G Katze, Katrina M Waters

**Affiliations:** 1 Computational Biology and Bioinformatics Group, Pacific Northwest National Laboratory, Richland, Washington, USA; 2 Department of Pathobiological Sciences, Influenza Research Institute, University of Wisconsin-Madison, Madison, Wisconsin, USA; 3 Department of Microbiology, University of Washington, Seattle, Washington, USA; 4 Oregon Health and Science University, Division of Biostatistics, Department of Public Health and Preventive Medicine, Portland, Oregon, USA; 5 Oregon Health and Science University, Knight Cancer Institute, Portland, Oregon, USA; 6 Battelle, Columbus, Ohio, USA; 7 Division of Virology, Department of Microbiology and Immunology, Institute of Medical Science, University of Tokyo, Tokyo 108-8639, Japan; 8 Department of Special Pathogens, International Research Center for Infectious Diseases, Institute of Medical Science, University of Tokyo, 108-8639, Japan; 9 ERATO Infection-Induced Host Responses Project, Saitama 332-0012, Japan; 10 Washington National Primate Research Center, University of Washington, Seattle, Washington, USA

**Keywords:** systems biology, influenza infection, host response, network inference, comparative transcriptomics

## Abstract

**Background:**

Understanding host response to influenza virus infection will facilitate development of better diagnoses and therapeutic interventions. Several different experimental models have been used as a proxy for human infection, including cell cultures derived from human cells, mice, and non-human primates. Each of these systems has been studied extensively in isolation, but little effort has been directed toward systematically characterizing the conservation of host response on a global level beyond known immune signaling cascades.

**Results:**

In the present study, we employed a multivariate modeling approach to characterize and compare the transcriptional regulatory networks between these three model systems after infection with a highly pathogenic avian influenza virus of the H5N1 subtype. Using this approach we identified functions and pathways that display similar behavior and/or regulation including the well-studied impact on the interferon response and the inflammasome. Our results also suggest a primary response role for airway epithelial cells in initiating hypercytokinemia, which is thought to contribute to the pathogenesis of H5N1 viruses. We further demonstrate that we can use a transcriptional regulatory model from the human cell culture data to make highly accurate predictions about the behavior of important components of the innate immune system in tissues from whole organisms.

**Conclusions:**

This is the first demonstration of a global regulatory network modeling conserved host response between *in vitro *and *in vivo *models.

## Background

The 1918 influenza virus pandemic was one of the most devastating in history, and is estimated to have killed over 50 million people worldwide [1]. The continued circulation of highly pathogenic avian H5N1 viruses and the emergence of the 2009 H1N1 pandemic virus has revived concerns about another lethal pandemic [2,3]. Although H5N1 viruses are largely zoonotic, human infections have occurred, with mortality approaching 60% [4], and there have been reports of limited human-to-human transmission [5-8]. Thus, there is a considerable need to understand the processes that drive pathogenicity of influenza, both in terms of viral dynamics and the host response to infection.

The selection of the appropriate model to study viral pathogenicity is essential to maintain relevance with human disease. Given their considerable similarity to humans, macaques are an excellent choice for studying the host response to influenza infection [9]. However, they are expensive, genetically diverse, and not amenable to certain types of downstream perturbation analysis (i.e. genetic deletions of host response genes). Influenza viruses also can infect and induce pathogenicity in mice, but mice are not a natural host. Indeed, comparison of H5N1 infections between mouse and macaque systems has shown that some aspects of the host response are not entirely conserved [9,10]. *In vitro *cell culture systems have been used extensively to study influenza infection [11] and have provided many insights into viral dynamics and host response. However, cells in culture exist in a highly artificial environment, which does not include any of the complex interactions between cell types that dynamically alter the environment and induce corresponding responses in the cell.

Profiling gene expression using microarrays has been used extensively for characterizing the host response to influenza infection [12-17]. Many of these studies have compared different viral strains or mutants within in the same experimental model, but few have directly compared the same virus between model systems. In fact, there is a general paucity of studies that compare similar stimuli in different systems, especially with regard to *in vitro *and *in vivo *models. One of the difficulties confronting such studies is that processes occur on different time scales in different models, and so simply comparing responses between systems is not a straightforward exercise.

The aim of our current work is to determine the extent to which transcriptional regulation of the host response during influenza infection is conserved between *in vitro *and *in vivo *models, as well as across species. In doing so our goal is to provide a computational framework for assessing similarities between experimental systems. Three complementary approaches are employed. An approach to compare dynamics at a functional level, an approach that identifies conserved patterns of coexpression, and an inference method that identifies conserved regulatory influences between systems. We show significant functional, coexpression and regulatory similarities between *in vitro *cell culture and animal models responding to infection with influenza. An important point is that none of these approaches rely on matching comparable time points between experiments, and thus we use them to compare data from different systems with very different sampling times and dynamics. We identify a number of conserved processes, including those involved in hypercytokinemia that have been previously identified as mediators of pathogenesis in H5N1 infection, and implicate novel genes in pathogenesis. For the first time we show how computational analyses can be used to reconcile data from cell culture and whole animal models of influenza infection to gain insight into the biological responses to infection and their dynamics in different systems.

## Results

### Overall approach

To compare host response to highly pathogenic avian influenza virus infection, we examined global gene expression response in human bronchial epithelial cells (Calu-3 [18]), lungs of inbred mice, and lungs of macaques [12] infected with influenza A/Vietnam/1203/2004 (referred to here as VN1203). This strain of influenza was isolated from a fatal human infection, and has been previously reported to be lethal in mice and at least partially lethal in macaques [12]. Previously, experiments were performed to follow the course of VN1203 infection by microarray analysis in Calu-3 cells at six time points post-infection up to 24 hours [19], and in macaques on days 1, 2, 4, and 7 days post infection (p.i.) along with a seasonal H1N1 strain (Tx91) with low pathogenesis and two reassortants of the 1918 virus [12]. Additionally, to examine the response to VN1203 in another important system, we generated microarray data from mice on days 1, 2, 4, and 7 days p.i. at three different dosages. Together, these studies provide a wealth of data about the host response to highly pathogenic VN1203 across three very different systems, both *in vitro *and *in vivo*. We note that though mouse and macaque are not known natural hosts for influenza, they are well-understood model systems that replicate many features of pathogenesis in other hosts.

To characterize the similarities between *in vitro *and *in vivo *models of influenza infection, we first used a functional similarity approach to compare the dynamics of response to infection in Calu-3 cells with that in macaques. This approach is useful because it does not rely on identification of homologs between the two species and makes use of functional information for non-homologs to determine similarity. We focused on comparison of infection in Calu-3 cells and macaque because we were primarily interested in determining the similarities and differences between *in vitro *and *in vivo *systems. We next compared the expression of homologs between Calu-3, mouse and macaque infections using an approach that determines similarities in co-expression patterns between systems. Finally, we used a network inference approach that employs multivariate regression and variable selection to formulate a model from the Calu-3 data that can predict the behavior of functional groups or individual genes in mice and macaques based on the expression levels of a small number of putative regulatory influences. To assess the ability of the Calu-3 model to provide information about more complex systems, we applied it to predict behavior in mouse and macaque. Comparison between different systems sampled at different time points is possible because our approaches do not require matching comparable time points between systems, a process that would be likely to introduce considerable bias to the results. In this study we aim to gain a better understanding of the conserved response to influenza across species and grant us additional information about the type of host responses to influenza *in vivo *that can be predicted or validated using *in vitro *experimentation.

### Conserved functional processes in host response to VN1203 in human Calu-3 cells and macaque

Comparisons of system responses in different organisms are complicated by the lack of one-to-one correspondence between genes in different species. To avoid this complication, we compared the response to VN1203 using a function-centric approach, in which genes are treated as members of different functional groups and the response to the stimulus is assessed at a functional level using gene ontology (GO) terms [20]. Comparisons between functional processes across systems can be informative about similarities and differences between responses at a higher level of abstraction. Such a comparative analysis is made possible by the fact that genes from different species are annotated using a common set of GO terms. By identifying functional groups that behave similarly between different organisms we can begin to focus on processes common to response and to get a better overall indication of how different organisms respond to the same virus.

**Figure 1:**
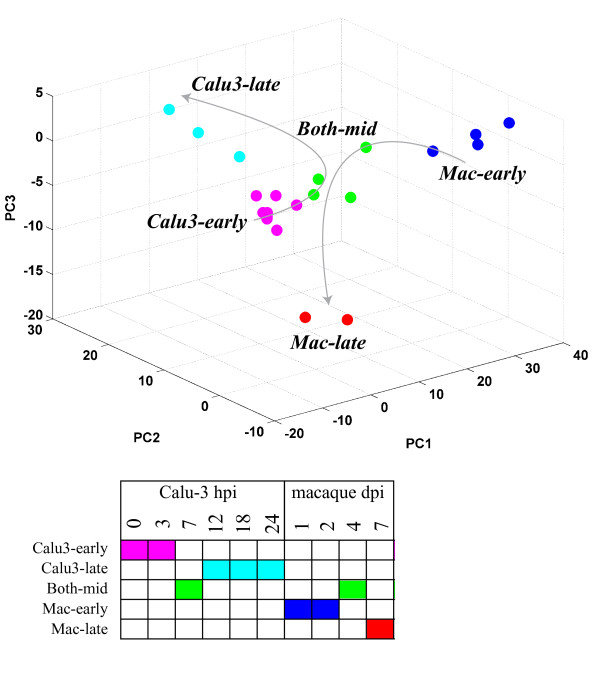
**Functional similarity between VN1203-infected Calu-3 cells and macaques**. Differentially expressed genes in each treatment were analyzed for their functional enrichment in GO biological process terms. Treatments (system, virus and time combinations) were clustered together based on the functional enrichment scores for a set of 76 GO terms using hierarchical clustering. A partitioning that results in 5 clusters is illustrated using distinct colors for each of the clusters. Treatments are projected on to the first three coordinates obtained via principal component analysis of the matrix of enrichment scores, and are colored based on their cluster membership. Lines indicate the temporal progression of VN1203 infection in each system. Treatments present in each cluster are listed at right.

To identify functional groups and components of processes that are conserved and unique between the response in Calu-3 cell culture and lungs of macaques infected with VN1203 we computed the statistical enrichment for all GO biological process terms for differentially expressed genes (p < 0.05, fold-change > 1.5 compared to mock) for each treatment. Here, a 'treatment' refers to microarray data from a single host (Calu-3 or macaque), virus (VN1203 or a non-pathogenic H1N1 strain in macaque) and time post-infection. We have included the non-pathogenic H1N1 (Tx91 seasonal strain) infection data in macaque with the same time points as the VN1203 infection as a control to highlight what a minimal pathogenic response looks like in this analysis. A total of 211 GO terms were significant (p <= 1e-4) in at least one treatment looking across all treatment groups. We removed terms that were either too broad or too specific by selecting for terms with an information content score [21] between 5 and 7.5, and this resulted in a final list of 76 enriched biological process. In this application the information content represents a measure of how specific the functional category is in terms of number of genes it annotates. Next, we clustered the different treatments based on their enrichment scores [-log10(p)] for these 76 terms using hierarchical clustering. By projecting the enrichment scores of each treatment onto the principal component coordinates of the 76-dimensional process term space, we were able to partition the individual observations by treatment (Figure 1). These results indicate that the early time points from the macaque-VN1203 infection (days 1 and 2; *Mac-early*), the macaque-VN1203 late day time point (day 7; *Mac-late*), and the late time points from the Calu-3 VN1203 infection (12, 18 and 24 hour; *Calu3-late*) each formed separate clusters in the process term space. The 7 hour time point from the Calu-3 VN1203 infection, both 4 day replicates of the Macaque-VN1203 infection and one of the 4 day replicates from the Macaque-seasonal influenza virus infection cluster together (*Both-mid*). Interestingly, all of the early (0 and 3 hour) time points from the Calu-3 VN1203 infection form a separate cluster that included the Macaque-seasonal influenza virus infections spanning 1 to 7 days (*Calu3-early*). This analysis indicates that treatments clustered by functional similarity reflect the temporal progression through infection in both systems.

To obtain a concise view of the processes that were enriched in each of the treatment clusters, we condensed the list of 76 process terms to a set of 13 GO term groups using the semantic similarity between GO terms [22]. The geometric mean enrichment score for each treatment cluster-GO group combination is presented in Table 1. As seen in Table 1 the *Calu3-early *treatment cluster does not show strong enrichment in any of the processes (enrichment score >1.3 corresponds to p < 0.05), possibly because there are a small number of genes differentially regulated at these early time points. Processes related to viral response, chemotaxis, muscle contraction, and innate immunity were found to be highly enriched in all other treatment clusters. Some of these functional groups represent systems-level processes (e.g. muscle contraction), which are not relevant to the *in vitro *cell culture system. However, these larger functional groups reflect underlying cellular processes that can be attributed to individual genes, even in the *in vitro *system. The *Calu3-late *cluster is highly enriched in processes related to translation, regulation of immune response including cytokine production, cell adhesion, and cell division and mitosis that is similar to what was observed for both the *Mac-early *and *Mac-late *clusters. A unique feature of the *Calu3-late *cluster is its enrichment in protein degradation and anti-apoptosis. This may be indicative of the marked cytopathic effects at 18 and 24 hours post-infection (data not shown) in the Calu-3 cell cultures, and is a normal feature of programmed cell death processes [23]. The *Mac-early *and *Mac-late *clusters are both strongly enriched in processes related to leukocyte proliferation, indicating a key function of immune cells in the *in vivo *model. This response is not reflected in the *in vitro *transcription data and this is likely due to the nature of the cell culture system, which contains only epithelial cells. Interestingly, both these clusters are enriched in very similar sets of functional groups, though with different levels of significance. The different significance levels reflect different numbers of genes annotated with these categories and so indicate the differences present between the two clusters. Overall, these data suggest that there are conserved aspects of response to VN1203 at a functional level between the Calu-3 cell system and macaques, despite the difference in the dynamics of these responses between *in vitro *and *in vivo *systems.

**Table 1:** Functional similarity between *in vitro *and *in vivo *models of influenza infection.

Biological Process	Enrichment Score^a^				
	Calu3-early	Calu3-late	Both-mid	Mac-early	Mac-late
muscle contraction/circulatory system process	0.16	1.39*	**2.23**	**1.40**	**2.03**
chemotaxis	0.18	**2.62**	**5.01**	**11.07**	**6.16**
response to virus/innate immune response	0.24	**2.06**	**2.54**	**7.78**	**2.96**
response to external stimulus/locomotion	0.29	**2.03**	**2.83**	**6.31**	**2.43**
muscle system process/smell perception	0.20	0.95	**1.49**	**1.75**	**2.92**

mRNA processing/translation	0.18	**3.96**	0.73	**1.47**	**1.85**
regulation of immune response	0.21	**1.69**	1.04	**3.54**	**1.57**
mitosis/cell cycle phase	0.24	**1.90**	0.78	**1.54**	**2.69**

cytokine production/secretion	0.19	**1.73**	0.80	**1.98**	1.05
cell adhesion/division/microtubule-based process	0.30	**1.99**	0.80	0.95	**2.00**

transcription reguln/protein degradation/anti-apoptosis	0.17	**2.73**	0.62	1.07	0.88
proteasomal protein catabolic process	0.23	**3.34**	0.69	0.98	0.93

regulation of leukocyte proliferation	0.18	1.09	0.73	**3.68**	**1.46**

^a ^The geometric mean enrichment score was calculated for each treatment cluster-GO group combination. Enrichment score >1.3 corresponds to p < 0.05.

*bold type indicates statistically significant values

### Conserved expression dynamics of response to influenza virus infection in human Calu-3 cells, mouse and macaque

We were interested in investigating the correlation of functional responses among the Calu-3 cells, and macaque and mouse on a gene-by-gene basis, but were faced with the fundamental problem that the time scales between the *in vitro *and *in vivo *studies were very different. A traditional correlation measure (for example, Pearson correlation) between gene expression profiles in both datasets can be used if the conditions are matched with each other (here called inter-correlation), however this does not account for genes that may have different dynamics in both systems or comparisons for which the time points are different. To address these limitations we developed an alternative approach, called cross-coexpression analysis, which can identify groups of genes that have similar expression patterns and are likely to be co-regulated but display different temporal dynamics or dynamic range, the minimum and maximum levels measured, caused by drift in the technology platform.

For cross-coexpression analysis, we constructed a coexpression matrix for each dataset independently by calculating the correlation between the expression profiles for all pairs of genes. We define the cross-coexpression matrix as the mean of the individual matrices, and pairs of genes in this matrix with values close to 1 are highly correlated in both datasets. We then applied a background correction in which the cross-coexpression matrix was normalized against a set of randomized matrices. This normalized matrix was used to calculate Z scores for the cross-coexpression values. In this matrix, pairs of genes that have high mean correlation values are considered to be coexpressed in each dataset. This does not mean that the pair of genes necessarily exhibit the same expression pattern in all three datasets; they may have different dynamics, magnitude and directionality of changes in each organism, but remain highly correlated to each other in the organisms independently.

To ascertain how much extra information could be obtained using our cross-coexpression approach versus traditional comparative methods, we initially compared the number of genes that could be identified as having coordinated regulation across two systems. For this analysis we compared VN1203 infection in mouse (MOI 10^4^) and macaque because there are identical time points (see Additional Files 1 and 2). This analysis showed that only 9% of homologs could be shown to have highly correlated expression profiles between systems, but that 98% of the homologs display coordinated behavior in both systems. Coexpression can provide an estimate of true co-regulation, as more observations of the system are added the prediction of co-regulated genes becomes more confident. Since the original macaque experiment included several strains of influenza, we included data from these strains to provide better discrimination of co-regulated genes. Our final analysis therefore includes 104 observations of the system (including biological replicates for macaque and mouse); the Calu-3 cell infection data (two sets of 6 time points), data from each viral strain in macaque (four time points, four viruses, two replicates), and each dosage in mouse (four time points, three dosages, five replicates). This comparison revealed that 64% of homologs displayed coordinated behavior in each system. This is a significant finding because it allows detection of coordinated transcripts between experiments, which have different time points, viral dosages, and strain differences.

**Figure 2:**
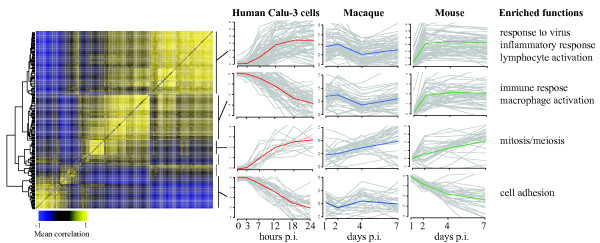
**Cross-coexpression analysis of avian influenza response in Calu-3, mouse and macaque**. The heatmap shows the cross-coexpression matrix of homologs in the Calu-3, mouse and macaque datasets representing the mean coexpression of each pair of genes (columns indicate one gene of a pair and rows indicate the other) from all three sets. A dendrogram from hierarchical clustering is shown at left and was used to divide the genes into six clusters (indicated by bars at right). Plots show the expression levels (log_2 _fold-change versus mock infected) of all genes in each of the four largest clusters (grey lines) and cluster means are represented by colored lines for Calu-3 (red), macaque (blue) and mouse (green). Significantly enriched functions for each cluster are listed on the right. This figure shows that our cross-coexpression analysis identifies groups of genes that are coexpressed in each organism but have different dynamics in each.

To identify functions enriched in cross-coexpressed groups of genes, we clustered the combined correlation matrix (cross-coexpression matrix) using hierarchical clustering and then performed functional enrichment on the resulting clusters. Figure 2 shows a matrix of the subset of transcripts with high correlation values with at least one other transcript as a heatmap, identification of six clusters based on this heatmap, functional labels for the clusters, and expression profiles of the four largest clusters in Calu-3 cells, mouse, and macaque for VN1203 infection. The transcripts in the shared clusters and functional enrichment for the clusters are provided in Additional File 3.

One of the four clusters in Figure 2 shows similar patterns and is enriched in mitosis and meiosis processes. In contrast, the other clusters display different dynamics in overall response across the three systems. There are two clusters that are enriched in immune response functions, and each is consistently regulated in mouse and macaque, but they show differential regulation in the Calu-3 cells. The response to virus cluster (top in Figure 2) is upregulated over time, but the more general immune response cluster is down-regulated. The general immune response cluster contains the *CASP1 *and *PYCARD/ASC *genes, two of the three components of the inflammasome, a multi-protein complex responsible for activation of the inflammatory response [24]. The third component, *NLRP3 *is not identified in this analysis since it was not classified as differentially regulated in the mouse experiment, but it shows a similar pattern of expression as this cluster. The functions identified in these clusters are similar to those identified in our functional analysis, showing that the two approaches are complementary. Importantly, the cross-coexpression analysis allows identification of the dynamic trends of the groups of transcripts in each dataset that comprise these functional groups, which was not possible using the functional analysis approach.

### Modeling regulatory influences involved in response to influenza infection across systems

The results from our functional comparison analysis and cross-coexpression analysis show that there are limited but significant similarities in the transcriptomic response to influenza infection in a human cell line, and mouse and primate model systems. We next applied a predictive transcriptomic modeling approach to determine if data from the human cell culture system can predict behavior in an *in vivo *system. We then used the model to identify the best regulatory predictions across the three data sets.

**Figure 3:**
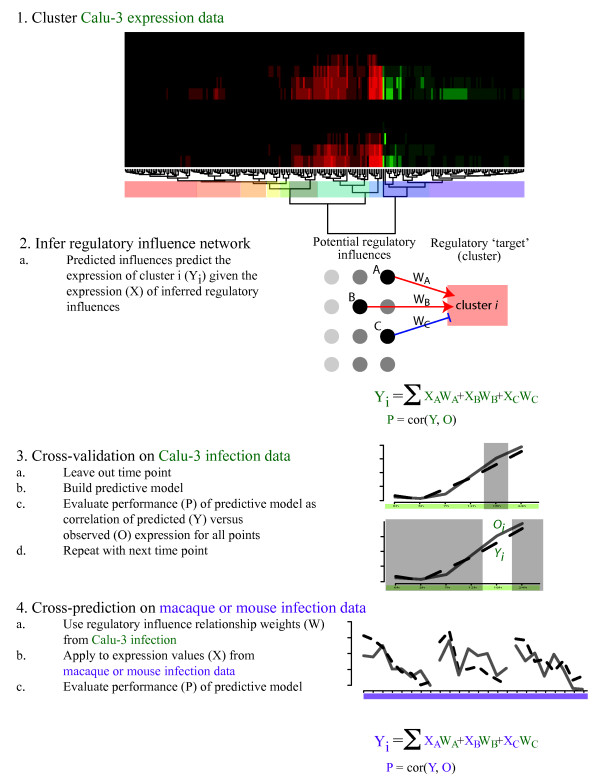
**Overview of cross-predictive modeling approach**. To allow cross-predictive comparison of response to influenza infection between Calu-3 and *in vivo *systems, we first clustered the Calu-3 expression data (1) from two similar experiments using hierarchical clustering. The clusters (colored boxes) are used to summarize system behavior and serve as the 'targets' for inference. A regulatory influence network is inferred (2) that relates the expression of inferred regulatory influences (X) to the mean expression (Y) of each target cluster (i). Cross-validation (3) is carried out by leaving out expression data from each time point in turn, inferring a model, then using the model to predict the behavior of each cluster for the left out time point. Performance of the model is assessed as the gene-weighted mean correlation between the predicted (Y) and observed (O) expression of all clusters. Finally, the weights from the Calu-3 model are applied to the macaque/mouse data and performance assessed by evaluating the gene-weighted mean correlation between the predicted expression and the observed expression in macaque/mouse for each cluster.

The modeling approach that was used is based on a previously published multivariate regression method [25,26] that we have successfully applied to eukaryotic systems [27,28]. In this approach multivariate regression with variable selection using the lasso algorithm [29] is used to learn relationships between the expression levels of regulators and groups of genes from a set of transcriptomic measurements (e.g. a time course response), which are the targets of the regulators. Using this approach, we can make predictions about which regulatory influences may be responsible for the behavior of co-regulated gene groups and generate a model that can be used to predict the behavior of co-expressed groups under novel conditions. We initially inferred a regulatory influence model using two Calu-3 expression datasets (Figure 3). We then evaluated model performance using a cross-validation approach in which multiple models are inferred from datasets with one time point held out. The performance of the model was then evaluated as the correlation of the predicted expression value for that cluster (Y) with the observed expression value (O) for the excluded time point.

**Figure 4:**
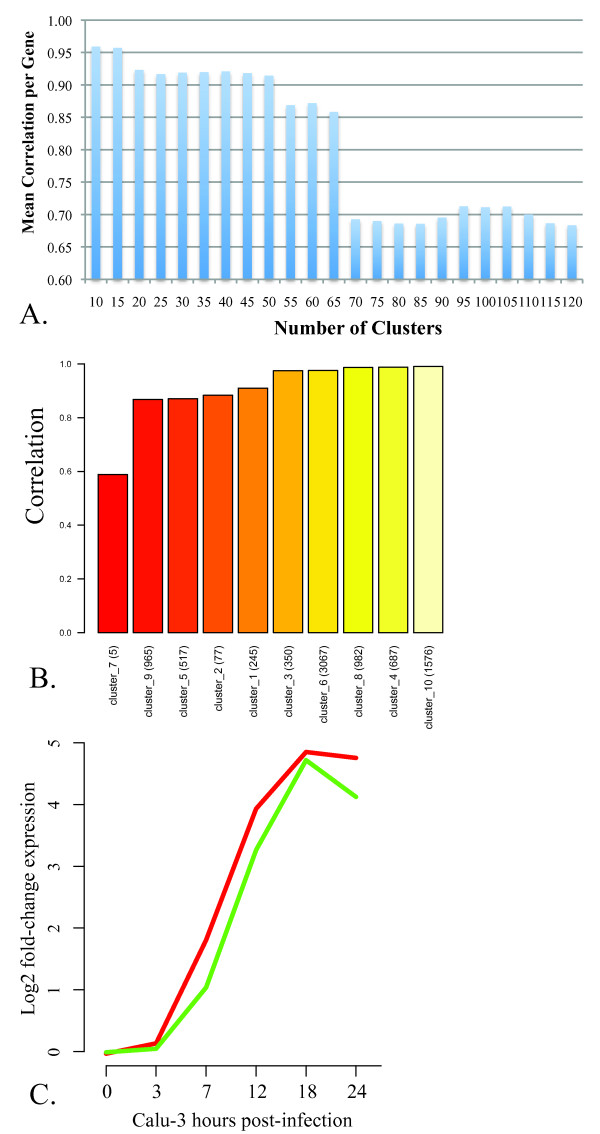
**Performance of Calu-3 regulatory influence model in cross-validation**. **A. Performance of inferred models with varying numbers of target clusters**. The cross-validation approach described was used to infer models based on varying numbers of target clusters (X axis) from the Calu-3 response to avian influenza infection. Performance is expressed as the mean correlation (Y axis) of predicted expression to the observed expression normalized to the number of genes in each target. **B. Performance inferred model at predicting expression of co-expressed clusters**. The cross-validation approach described was used to infer a model based on ten co-expressed clusters (X axis) from the Calu-3 response to avian influenza infection. Performance is expressed as the mean correlation (Y axis) of predicted expression to the observed expression for each cross-validated time point. Details about each cluster are provided in Table 2. **C. Predicted and observed expression patterns for an innate immune-related cluster**. The predicted expression levels (green line) from cross-validation are shown over the six time points post-infection versus the observed mean expression (red line) for cluster 8 (see Table 2).

Using all the differentially expressed transcripts (8471) from the dataset of Calu-3 cells infected with VN1203, we evaluated the overall performance of a series of cross-validated models using varying numbers of clusters defined by hierarchical clustering, from 5-120. We found that maximal performance was achieved with 10 or 15 clusters (Figure 4A), and chose to focus on the simpler 10 cluster model as the base for our predictive modeling. The gene-normalized mean correlation between the predicted expression profiles and observed expression profiles over all time points was 0.96. We showed overall good performance (Y axis) of most target clusters (categories), ordered by performance in Figure 4B. In Table 2 we provide a list of the functional categories that are statistically enriched in each of these 10 clusters, as well as the performance of the model for each cluster (Calu-3 column). To ascertain whether these results are likely to have arisen by chance we randomly permuted the cluster membership for each cluster 25 times and used this information to calculate a p-value. Statistically significant (p-value < 0.05) performance results are indicated in Table 2 with an asterisk. Figure 4C shows the performance of an early up-regulated cluster (cluster 8) in the two experiments. The relative expression levels (Y axis) predicted by the model (green line) are shown compared to the mean gene expression from the cluster (red line), for each of the time points in one experiment (X axis). This plot shows that the predictions, made using cross-validation, are very close to the observed expression in this simple time course which suggested that the overall performance of this model was very good based on cross-validation. The trained model was then applied to the macaque and mouse data to ascertain which portions of the Calu-3 model are consistent with the regulator-target relationships in these *in vivo *systems.

**Table 2:** Functional enrichment of Calu-3 clusters.

Cluster	Top biological functions enrichment within each cluster			Predictive ability^a^		
	Functional Pathways	p-value^b^	# molecules	Calu-3	Macaque	Mouse
1	None			0.89*	0.23	0.98*

2	cell surface receptor linked signal transduction	1.56E-02	6	0.88*	-0.44	0.28
	activation of eukaryotic cells	1.56E-02	9			
	developmental process of antigen presenting cells	1.56E-02	5			
	aggregation of blood cells	3.09E-02	4			
	growth of leukocytes	3.09E-02	4			
	replication of virus	3.09E-02	6			

3	binding of blood cells	5.22E-07	16	0.96*	0.44	0.98*
	activation of granulocytes	5.28E-07	11			
	stimulation of normal cells	6.52E-07	13			
	inflammatory response	6.56E-07	23			
	maturation of leukocytes	6.98E-07	14			
	chemotaxis of cells	9.01E-07	20			
	activation of monocytes	9.65E-07	9			
	activation of phagocytes	9.65E-07	13			
	activation of T lymphocytes	1.09E-06	16			
	immune response	2.82E-06	28			

4	developmental process of blood cells	2.38E-04	41	0.99*	-0.13	-0.99*
	function of lymphatic system cells	1.95E-03	5			
	recruitment of normal cells	4.40E-03	14			
	differentiation of lymphocytes	4.47E-03	20			
	function of leukocytes (including function of granulocytes)	5.51E-03	9			
	quantity of leukocytes	5.76E-03	25			

5	development of intercellular junctions	8.80E-04	10	0.83	-0.21	0.92*

6	cell division process	2.98E-03	253	0.96	0.25	-0.97*
	metabolism of carbohydrate	3.52E-02	80			
	transactivation	3.52E-02	127			
	cell death of connective tissue cells	3.83E-02	66			
	modification of DNA	5.02E-02	58			

7	secretion of cytokine/recognition of cells	2.97E-02	1	0.64	0.32	0.58
	methylation of protein or DNA	2.97E-02	1			

8	accumulation of calcium	3.13E-02	6	0.97*	0.68*	-0.86
	contraction of tissue	3.69E-02	16			
	neurotransmission	3.78E-02	20			
	blood pressure	3.78E-02	13			
	response of cells	3.78E-02	24			
	synthesis of cyclic AMP	3.78E-02	7			

9	None			0.72	0.25	0.86

10	transcription	1.67E-08	150	0.99*	0.72*	0.33
	protein kinase cascade (including IKKB/NFkB cascades)	4.25E-03	33			
	activation of cyclin-dependent protein kinase	5.06E-03	11			
	developmental process of organism	1.62E-02	81			
	cell cycle progression	2.79E-02	67			
	activation of protein	4.00E-02	20			

^a ^asterisk indicates statistical significance versus random for predictive ability

^b ^Benjamini-Hochberg adjusted

### Macaque response to infection with influenza can be predicted using a Calu-3 regulatory model

We next explored what portions of the host response to influenza *in vitro *were predictive of transcriptomic behavior *in vivo *using the regulatory model trained on Calu-3 data to predict expression data from macaque lungs. As the case with the cross-coexpression analysis above, we included data from macaque infections with VN1203 as well as other strains of influenza (H1N1, see Methods) in order to increase confidence that our results represent real regulatory influence relationships. We filtered the Calu-3 model to include inferred regulators present in macaque and genes in target clusters present in macaques. Regulators not present in macaque are ignored for predictions (see Methods) and expression of target clusters is calculated as the mean expression of genes present in macaque, ignoring non-homologs. We evaluated the models generated from the Calu-3 with different numbers of clusters (5-120), and as in the case of our cross-validation, found that the model containing ten clusters had the best cross-prediction performance with a correlation of 0.39. The results of the cross-predictions suggest that although most of the clusters were not well-predicted in macaque (Table 2; Macaque column; membership of all DE genes found in Additional File 4), clusters 8, and 10, show statistically significant correlations, 0.68 and 0.72, respectively (Table 2), versus randomly permuted clusters. These two clusters represent 982 and 1576 genes, respectively, accounting for approximately 30% of all differentially expressed transcripts. The functional enrichment of these two clusters (Table 2) is dominated by immune response and regulation of transcription processes, similar to our functional comparison in Table 1. It is interesting, however, that cluster 2, which represents T-cell activation and interleukin-2 production is negatively correlated with predictions, suggesting that these processes are regulated differently between macaque lung and Calu-3 cells, though this is not statistically significant. The same is true for clusters 4 and 5, which contains negative regulators of transcription, inflammatory response and cell migration processes. Numbers of genes and mouse and macaque homologs in each cluster are listed in Additional File 5.

### Calu-3 regulatory model predicts mouse response to influenza at different dosages

To assess how consistent the model derived from human cell culture experiment would be with the host response to influenza in a different animal model, we next evaluated the performance of the Calu-3 model on data from inbred mice. In the mouse experiment three different dosages of V1203 were used to infect groups of mice, and high correlation results from predictions would indicate that our model is capturing dose differences across the data set. Because the numbers of differentially expressed homologs were significantly lower than in macaque (Additional File 5) we examined the significance of the performance value for each cluster relative to 25 random permutations of genes for that cluster. Table 2 indicates significant performance results with an asterisk (Mouse column). From this analysis were able to detect both similarities and differences in response to the virus across models. Clusters 1, 5 and 9 accurately predicted the mouse data but not as well in the macaque data. Interestingly, these three clusters have few enriched functions, indicating that they may represent a diverse set of responses. The results for cluster 3 are notable as it represents portions of the immune response that are conserved in all three systems, and in this case the predictions in macaque are reasonable (0.44 correlation), but predictions in the mouse are much better. We further found that our model correctly identified the trend in overall expression between doses for those clusters that are significantly predicted (data not shown). This further supports the notion that a Calu-3 model may be able to accurately predict some regulatory influences on influenza response. It is also interesting that clusters 4, 6, and 8 are highly anti-correlated in their regulatory predictions in mouse, reflecting significant differences between species. In all cases with the mouse clusters the number of homologous genes is low (see Additional File 5) and few regulatory influences used for the model were identified as homologous, suggesting that these results should be taken as preliminary. However, the fact that these clusters perform significantly better than the random controls provides support for our results.

### Prediction of important immunological responses in macaque and mouse

Our regulatory models suggest conserved regulation between the Calu-3, mouse, and macaque, following closely our results from function-centric and cross-coexpression analyses. To develop a model with finer-grained detail about groups of genes being commonly regulated, we identified the best predictions made by the Calu-3 model to the macaque data from a large number of possible cluster sizes using hierarchical clustering (see Additional Files 1 and 6). To reduce the likelihood of false positives, we randomly clustered genes 25 times and used this to provide a significance estimate for predictions of individual genes. Using this method, we identified a group of top scoring gene predictions that are unlikely to have occurred by chance (Table 3).

**Table 3:** Genes exhibiting consistent relationships with inferred regulatory influences between the Calu-3 model, macaque, and mouse response data.

Symbol	ID	Description	Macaque^a^	Mouse
ABI3	NM_016428	ABI gene family, member 3	**0.35**	
MXD1	NM_002357	MAX dimerization protein 1	**0.35**	0.53
ALOX5	NM_000698	arachidonate 5-lipoxygenase	**0.36**	
COL4A3	NM_000091	collagen, type IV, alpha 3 (Goodpasture antigen)	**0.36**	
C1QTNF3	NM_181435	C1q and tumor necrosis factor related protein 3	**0.38**	
CH25H	NM_003956	cholesterol 25-hydroxylase	**0.38**	**0.86**
FOS	NM_005252	v-fos FBJ murine osteosarcoma viral oncogene homolog	**0.39**	
TNFSF13B	NM_006573	tumor necrosis factor (ligand) superfamily, member 13b	**0.40**	
IL1R2	NM_004633	interleukin 1 receptor, type II	**0.40**	**0.88**
CD86	NM_006889	CD86 molecule	**0.40**	**0.87**
TNFAIP3	NM_006290	tumor necrosis factor, alpha-induced protein 3	**0.41**	0.70
ASCL2	NM_005170	achaete-scute complex homolog 2 (Drosophila)	**0.41**	
IRX4	NM_016358	iroquois homeobox 4	**0.41**	
BATF2	NM_138456	basic leucine zipper transcription factor, ATF-like 2	**0.41**	**0.88**
SCNN1G	X87160	sodium channel, nonvoltage-gated 1, gamma	**0.42**	
PARP11	NM_020367	poly (ADP-ribose) polymerase family, member 11	**0.42**	0.83
CMTM2	NM_144673	CKLF-like MARVEL transmembrane domain containing 2	**0.42**	
ADM	NM_001124	Adrenomedullin	**0.43**	0.77
PRSS12	NM_003619	protease, serine, 12 (neurotrypsin, motopsin)	**0.44**	
USP18	NM_017414	ubiquitin specific peptidase 18	**0.48**	**0.94**
PI3	NM_002638	peptidase inhibitor 3, skin-derived (SKALP)	**0.48**	
IL29	NM_172140	interleukin 29 (interferon, lambda 1)	**0.54**	
UPP1	NM_181597	uridine phosphorylase 1	**0.57**	0.84
INDO	NM_002164	indoleamine-pyrrole 2,3 dioxygenase	**0.58**	**0.90**
LDHC	NM_002301	lactate dehydrogenase C	**0.58**	
CXCL10	NM_001565	chemokine (C-X-C motif) ligand 10	**0.58**	**0.96**
RND1	NM_014470	Rho family GTPase 1	**0.59**	**0.85**
IFIT2	NM_001547	interferon-induced protein with tetratricopeptide repeats 2	**0.60**	**0.97**
IFIT1	NM_001548	interferon-induced protein with tetratricopeptide repeats 1	**0.62**	**0.96**
IFIT3	NM_001549	interferon-induced protein with tetratricopeptide repeats 3	**0.64**	**0.97**
ATF3	NM_004024	activating transcription factor 3	**0.66**	**0.86**
OASL	NM_003733	2'-5'-oligoadenylate synthetase-like	**0.67**	
CCL4	NM_002984	chemokine (C-C motif) ligand 4	**0.67**	**0.92**
JUNB	NM_002229	jun B proto-oncogene	**0.68**	0.66
MX2	NM_002463	myxovirus (influenza virus) resistance 2	**0.69**	**0.90**
XAF1	NM_017523	XIAP associated factor-1	**0.70**	
IL6	NM_000600	interleukin 6 (interferon, beta 2)	**0.71**	**0.92**
RSAD2	NM_080657	radical S-adenosyl methionine domain containing 2	**0.89**	**0.85**
OAS2	NM_016817	2'-5'-oligoadenylate synthetase 2, 69/71kDa	**0.92**	0.84

^a ^Xpred scores from Calu-3 model cross-predictions in macaque and mouse. Bold indicates statistical significance (p-value < 0.05)

Table 3 shows the genes that have the most consistent relationships with their inferred regulators from Calu-3 to macaque and Calu-3 to mouse. This table lists the gene symbol, identifier and description of genes with high cross-prediction scores (Xpred score; see Methods) to macaque relative to the randomized background (bold indicates statistical significance p-value < 0.05). The Xpred score indicates how well the behavior of individual genes is predicted across organisms. We also show the Xpred scores for prediction to the mouse infection data for those genes with differentially expressed homologs in mouse. This group represents the transcripts that appear to be regulated in similar ways in Calu-3 and macaque, presenting a hypothesis that the same regulatory influences are responsible for regulation of similar genes or sets of genes in both organisms.

**Figure 5:**
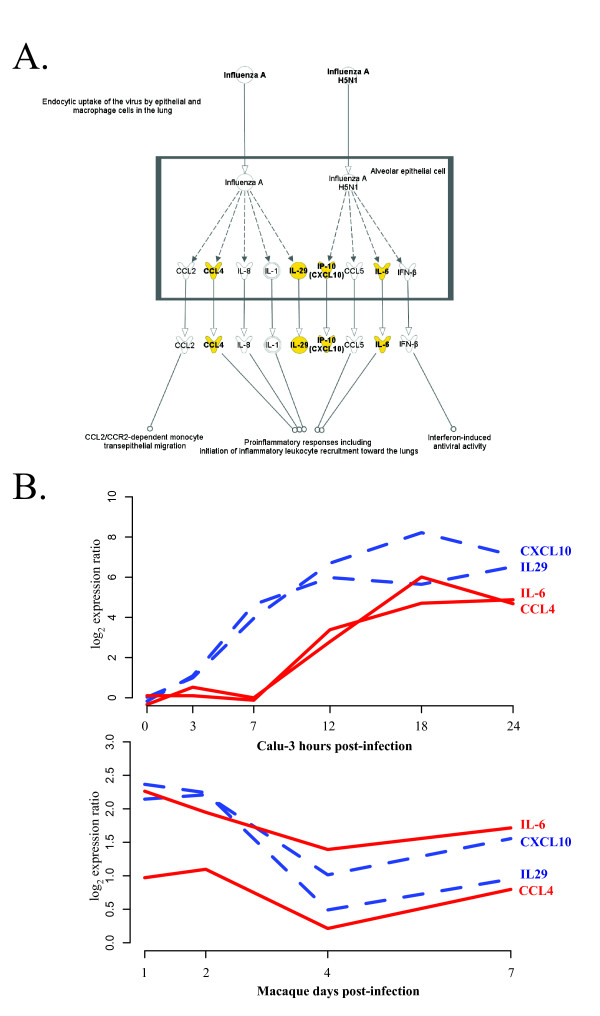
**Top Canonical Pathway: Role of hypercytokinemia and hyperchemokinemia in influenza**. **A**. Integrated Pathway Analysis (IPA) canonical pathway map of the most significant pathway enriched in the set of highly predicted genes (Table 3). Shown in yellow are the highly cross-predicted genes. **B**. Expression patterns of highly predicted genes in Calu-3 cells and macaques. Dashed blue lines (CXCL10, IL29) indicate an earlier response and red lines (IL6, CCL4) are later in the Calu-3 infection.

We next used Ingenuity Pathway Analysis (IPA; Ingenuity Systems) to determine the biological functions most significantly associated with this highly predicted set of genes. In Additional File 7 we list the most significant biological functions enriched within this highly predicted gene set as identified by IPA. This functional analysis again suggests a conservation of immunomodulatory pathways including growth-, metabolism-, and inflammatory- related genes. It is interesting to note that among the top functional pathways, we found that many of the highly predicted genes play roles in the mediation of multiple aspects of response. IPA was next used to determine the top canonical pathways represented within these highly predicted genes. These analyses indicated a highly statistical enrichment of genes within the hypercytokinemia and hyperchemokinemia in influenza pathway (Figure 5A). Hypercytokinemia is thought to play an important role in the pathogenesis of VN1203 [30,31]. The hypercytokinemia during influenza infection includes the over-expression of immune modulators including *CCL4, LI29, CXCL10 *and *IL6*. These molecules were identified in our analysis and their expression in Calu-3 cells and macaque lung cells during VN1203 infection is shown in Figure 5B. All of these genes show a very early response in Calu-3 cells beginning at hours 3-7 hours post-infection, indicating a robust, early response to VN1203 infection. Since our approach does not rely on matched time points between the different systems examined the results show the different dynamics of genes that are predicted to have similar regulation in both systems. This result indicates that the marked upregulation of these mediators is preserved in Calu-3 cells despite the notable absence of immune cell infiltration and that this transcriptional profile is predictive of cytokine overexpression in the whole lung of macaques.

Assessment of the relationships between these molecules using the IPA knowledge base illustrates that among the highly predictive genes we find a small network of molecules that were both directly and indirectly functionally related. This illustrates a coordinated prediction of regulators of chemotactic and inflammatory response such as *IL6 *and *CXCL10*, interferon and antiviral response (Additional File 8). The conserved regulation of upstream mediators of transcription may account in part for the conservation in cytokine transcript expression across species. This network also suggests the link between IL-6 gene expression and many other downstream mediators of response.

**Figure 6:**
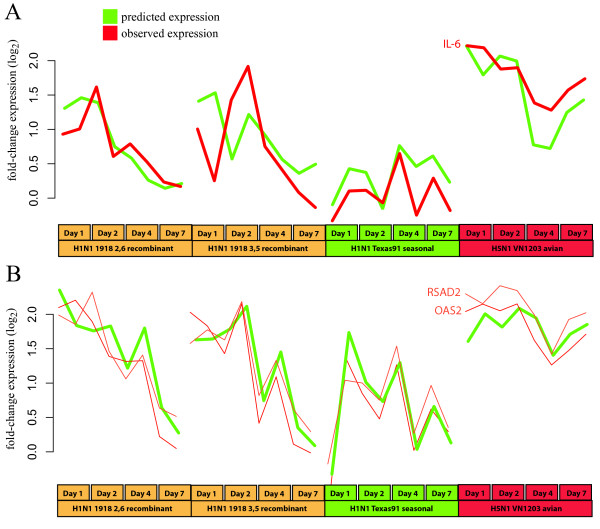
**Expression of highly predicted gene clusters**. **A**. The cross-prediction approach was taken to predict expression (green line) of the proinflammatory cytokine IL-6. The observed expression is shown as a red line. **B**. Behavior of RSAD2 and OASL in macaque based on the model inferred from Calu-3 expression data. This figure shows that the *in vitro *Calu-3 system can be highly informative about the behavior of certain genes in a whole macaque infection.

IL-6 is a cytokine that is a primary mediator of inflammatory response in influenza infection, and is involved in driving the hypercytokinemia response in VN1203 infection [30,31]. Figure 6A shows the expression of *IL6 *observed in the macaque study (red line) and predicted by our model trained in Calu-3 human epithelial cells (green line). The Calu-3 model is also capable of predicting IL-6 expression in the mouse system with a high degree of accuracy (Additional File 9).

In addition to the factors known to play a role in hypercytokinemia, this analysis also identified several other regulators of host response that have been less well characterized with respect to influenza including *XAF1, ATF3, ALOX5*, and *CH25H*. All these genes, except *ALOX5*, are upregulated during infection with VN1203. *XAF1 *is a pro-apoptotic factor that works by inhibiting the anti-apoptotic XIAP protein [32]. Apoptosis has been suggested to be a factor in the pathogenesis of influenza-induced encephalopathy [33], as well as pathogenesis in the lung and lung epithelium [12,34,35]. The transcription factor ATF-3 has been shown to be involved in apoptosis and cell cycle regulation, though its role as a pro- or anti- apoptotic factor is unclear [36]. It was also found to negatively regulate TLR signaling pathways in influenza infection [37], and *ATF-3 *-/- mice were more susceptible to hypercytokinemia [36]. Additionally, the arachidonate 5-lipoxygenase enzyme *ALOX5*, is downregulated in all systems and catalyzes an important step in the leukotriene synthesis pathway. Leukotrienes are important mediators of inflammation, but have not been extensively investigated with regard to influenza infection. Finally, the cholesterol 25-hydroxylase enzyme *CH25H*, regulates lipid metabolism and immune activation in response to interferon and is speculated to modulate the intensity of subsequent responses [38]. This raises the possibility that *CH25H *may be contributing to cytokine amplification by making the cells more sensitive to further stimulation. This offers a hypothesis for a possible mechanism of hypercytokinemia that can be further investigated.

Many of the predictions for these genes display minor inconsistencies with the observed profiles in the macaque data, but generally capture the trends in the data (data not shown). However, two genes, the 2'-5'-oligoadenylate synthetase paralog *OASL*, and the radical S-adenosyl methionine domain protein, *RSAD2*, are nearly perfectly predicted in the macaque. In Figure 6B we show the predicted expression pattern (green line) and observed expression patterns (red lines) for *OASL *and *RSAD2 *over the four different virus strains examined (X axis). As mentioned above, we did not specifically focus on the other viral strains in the macaque experiment with regard to their pathogenesis, but include the prediction results here to show support for the regulatory influences we have inferred. Both *OASL *and *RSAD2 *are interferon-induced genes and have been shown to have antiviral activity [39,40]. These genes represent a signature of a larger interferon response present in the highly predicted set of genes. This is supported by the presence of IRF1/2 transcription factor binding sites in the upstream regions of 16 of the 39 highly predicted genes, a significant enrichment (p-value 0) according to the cREMaG webserver [41]. The role of the interferon response in influenza infection has been extensively studied [30], but our finding that portions of the response are conserved between human cell culture and macaque infection models is novel.

### Predictive modeling reveals patterns of regulatory influence driving VN1203 response

One of the primary motivations in developing a predictive model of VN1203 response was to elucidate potential regulatory influences that drive host response. The group of highly predicted genes between the Calu-3 and macaque VN1203 response displayed similar patterns of expression in both systems, and in the mouse model. We next examined the predicted regulatory influences from our model. As described above, we chose a set of potential regulatory influences from genes annotated as transcription factors and those annotated as immune effectors from the differentially expressed genes used to construct the model. In Table 4 we show the top inferred regulatory influences for the highly cross-predicted gene list (from Table 3) in terms of numbers of genes predicted to be influenced. This analysis highlights several potential drivers of immune response to highly pathogenic influenza infection that are further discussed in the Discussion section, below.

**Table 4:** Top inferred regulatory influences for highly cross-predicted genes.

	Predicted Regulatory Influence^a^								
Gene Symbol	GTF2B	ATF4	IFI44	IFNGR2	FOXE1	BRF1	ELF1	ELF2	IRF2
**MXD1**	**-**	**+**				**-**	**+**		**-**
**C1QTNF3**	**+**		**-**	**-**	**-**				
**CH25H**	**-**	**+**				**-**	**+**		**-**
**FOS**	**-**			**+**					**-**
**TNFSF13B**			**+**	**+**					
**CD86**	**-**	**+**				**-**	**+**		**-**
**TNFAIP3**	**-**	**+**				**-**	**+**		**-**
**ASCL2**			**+**	**+**					
**IRX4**			**+**	**+**					
**BATF2**			**+**	**+**					
**PARP11**	**-**	**-**	**+**			**+**	**+**		
**CMTM2**			**+**	**+**					
**ADM**				**-**					**+**
**USP18**			**+**	**+**					
**PI3**	**-**	**-**			**+**			**-**	
**IL29**	**-**	**-**	**+**		**+**	**+**	**-**	**-**	
**UPP1**	**-**	**-**			**+**			**-**	
**INDO**	**-**	**-**	**+**						
**LDHC**	**-**	**-**			**+**			**-**	
**CXCL10**	**-**	**-**	**+**						
**RND1**	**-**			**+**					**-**
**IFIT2**	**-**	**-**	**+**		**+**	**+**		**-**	
**IFIT1**	**-**	**-**			**+**			**-**	
**IFIT3**	**-**	**-**			**+**			**-**	
**OASL**	**-**	**-**	**+**						
**CCL4**	**-**			**+**					**-**
**MX2**	**-**	**-**	**+**						
**XAF1**	**-**	**-**	**+**						
**IL6**	**-**			**+**					**-**
**RSAD2**	**-**	**-**	**+**	**+**		**-**			
**OAS2**	**-**	**-**	**+**	**+**		**-**			
***Count***	29	23	20	17	12	11	10	11	10
***Trend***	**-**	**-**	**+**	**+**	**+**	**-**	**+**	**-**	**-**

**^a ^**"+" indicates positive and "-" negative regulation. Genes that are only regulated by one of these regulators are not shown.

## Discussion

In this study we have used several different but complementary approaches to characterize the similarities and differences in the transcriptomic response of an *in vitro *human cell culture, inbred mice, and the outbred cynomolgus macaque to infection with a highly pathogenic avian influenza virus. This analysis represents a significant advance in the study of complex systems using simple *in vitro *models. Our approach is novel in that it compares transcription between systems at the level of regulation, in addition to a direct comparison of up or down trends in expression in response to influenza infection. A key feature of our approach is that it does not require comparable time points to be matched between systems, which may be very difficult or impossible when comparing *in vitro *and *in vivo *models. The approach uses several methods to evaluate functional similarities, and putative coregulation patterns to define the similarities and differences between expression dynamics across organisms and between *in vitro *and *in vivo *models of influenza infection. Our combined analysis and predictive regulatory model indicate that portions of the regulatory structure are preserved from *in vitro *cell culture to whole animal response to VN1203 infection. Our results support the notion that lung epithelial cells are involved in initiating hypercytokinemia [9], which is a primary component of pathogenesis of VN1203.

Our first approach to characterizing the responses to influenza infection involved identifying similar functional categories in differentially expressed genes between systems (Figure 1 and Table 1). This method has the advantage of utilizing all the data available from each system as it does not require identification of homologs between the two organisms. Our results showed that the human Calu-3 cell line exhibited similar dynamics as macaque lungs in response to influenza, at least for a subset of functional categories. Of specific interest is the high level of agreement in biological process categories containing genes involved in the innate response to viral infection, cytokine production and secretion, and chemotaxis. We have previously shown a high level of correlation among specific subgroups of genes represented by these categories after infection with VN1203 in Calu-3 cells and mouse lungs [19]. Thus, here we extend these observations to the macaque, an *in vivo *model that may more closely reflect human disease associated with VN1203 infection. Because VN1203 pathogenicity is likely partially regulated by dysregulation of immune signaling and hypercytokinemia, the observed commonalities between the three model systems strongly suggest a role for airway epithelium in VN1203 pathogenesis.

We found that evaluating the correspondence between the mouse and macaque infections by simple correlation between identical time points identified a small number of genes that displayed similar behavior between the two systems. This approach could not be applied to compare the Calu-3 expression data, since this experiment used very different time points than did the macaque and mouse experiments. Our cross-coexpression approach, on the other hand identified a large number of gene pairs that were correlated in each data set independently, and this revealed that a substantial portion of the homologs shared between the three systems display coordinated behavior. This coordinated behavior, as seen in Figure 2, has different dynamics in the different systems in some cases. Note that we do not expect to see identical patterns of expression in each system for the clusters shown since our approach does not directly compare expression patterns between different systems, but rather identifies patterns of coexpression that are conserved. The observation that some groups of genes display similar coordinated response to the same stimuli in different organisms is expected but has rarely been demonstrated at this level of detail. In fact similarity of response is an assumption made by all studies performed in *in vitro *cell culture or model organisms. We have shown that in the case of human cell culture, mouse and macaque responding to avian influenza this assumption holds for certain sets of functions and genes. This presents a powerful tool to guide future studies since experiments can be designed to investigate responses that are most similar between cell culture and samples from multicellular tissues in whole organisms.

To further characterize the putative conserved regulation between the different systems we employed a regulatory network inference approach. In this approach we used a multivariate regression method with variable selection to establish the minimal set of regulatory influences whose expression levels maximally explain the behavior of the target cluster (Figures 3 and 4 and Table 2). We first inferred a model from the cell culture experiment data then applied it to the *in vivo *data sets. We found several clusters whose expression levels in macaque and mouse infection were accurately described by the model. In this approach the Calu-3 data was used to train the model, then the macaque and mouse lung tissue data was used to validate the model. We assessed the significance of this finding using randomized validation sets and found that portions of the model were validated in each of these datasets.

Though these results are promising, there are several caveats that must be recognized with this method. The first is that we have used clustering to identify groups of genes with coordinated expression, and these clusters are used as the targets in the model. It is certainly true that individual genes in each cluster may have expression patterns that differ significantly from the average behavior of the cluster. This means that the resulting model can be considered to be fairly coarse-grained. To provide a more fine-grained model we used an approach to attempt to focus more on the regulation of small groups of genes, and implement a performance measure that incorporates the agreement of the prediction with the specific gene in the cluster (Xpred score, Figure 6 and Table 3). In this approach we identify small groups of genes in the model for which we can very accurately predict expression behavior in the *in vivo *systems. Strikingly, these patterns capture both dose effects in the mouse experiment and strain differences in the macaque experiment.

Additionally, the Calu-3 experiment used to train the model is extremely limited in size and number of different perturbations. We have shown that even with this limitation we can achieve a surprising level of accuracy in our model and future inclusion of more diverse data from cell cultures is likely to improve our model considerably. We are currently generating transcriptomic data from Calu-3 cells infected with different viral mutants. These data represent significant perturbations of the system that will improve our ability to discriminate true causal regulatory relationships from false-positive correlations. Additionally, the results presented in this study can be used to guide which responses might be most informative to study in the Calu-3 cell system.

This method depends almost entirely on measurement of mRNA levels by microarrays. Therefore it is unlikely to provide good predictions unless the expression levels of the regulatory influences reflect the activity of the cognate protein (e.g. a transcription factor). If the activity of a transcription factor is regulated solely by phosphorylation, for example, this approach will not be able to identify it as a regulatory influence on its target genes. However, using our cross-validated performance metric allows identification of clusters and their regulatory influences for which this assumption holds.

It is certainly possible that some of the predicted regulatory influences may be false positive predictions, and may not be causal influences. We have used a cross-validation approach to ensure that the regression method is not over fitting the data, and identifying false-positives in this way. Additionally, evaluation of the model on an entirely different system (e.g. the mouse or macaque data) allows identification of regulator and targets that exhibit the same relationship in the other system. Though this still does not answer the question of causality, it strengthens the claim that the relationship is not spurious and the result of conditions in one experiment. Finally, we have included genes that encode proteins capable of exerting indirect downstream regulatory influence (e.g. immune effectors). Further experiments will have to be performed to validate these predictions and to determine those that are truly causal.

We showed that a model trained on data from the Calu-3 cell line infected with VN1203 could accurately predict expression of a number of genes in lung tissue of infected inbred mice and in outbred macaques (Table 3). One of these genes, *IL6*, encodes an important acute phase cytokine that mediates transcriptional upregulation of many pro-inflammatory genes. Humans infected with VN1203 viruses exhibit abnormally high levels of IL-6 in serum [42,43], and aberrant upregulation of IL-6 is observed in primary human alveolar and bronchial epithelial cells in response to VN1203 infection, relative to infection with a low pathogenicity H1N1 isolate [44]. Levels of IL-6 in the serum and epithelial tissues were shown to be significantly higher in macaques infected with VN1203 relative to those infected with reassortant H1N1 viruses possessing the HA/NA, or HA/NA/NS genes of the pandemic 1918 strain, reflecting the higher pathogenicity of VN1203 in this study [see Figure S2 in [12]]. Expression levels of the IL-6 gene correlate well with the levels of the cytokine in macaque epithelium [12]. Mice lacking the IL-6 gene still succumbed to lethality induced by a VN1203 virus, suggesting that IL-6 effects are not sufficient to induce VN1203 pathogenicity [45]. Since high *IL6 *expression seems to be particularly correlated with VN1203 pathogenesis our model, *IL6 *could be useful for predicting pathogenicity of viral strains and mutations using data generated in cell culture, though direct comparison of these results with responses to non-pathogenic virus infection will be required to accomplish this. Though the numbers of genes that could be accurately predicted is quite modest, many are known to be important components of the immune system and will be useful in understanding the relationship between pathogenesis in different model systems.

VN1203 pathogenesis may be due in part to the early and robust immune cell infiltration in the lung [9]. Our data show that the initial targets of infection, the airway epithelial cells, are secreting factors that facilitate this, i.e. cytokines and chemokines. The expression of genes involved in hypercytokinemia in cultured epithelial cells at very early time points (3-7 hours p.i.; see Figure 5B) is clearly reflected in their expression in lung tissue (highest levels at day 1), strengthening the notion that the epithelial response may be contributing to hypercytokinemia *in vivo*. An exacerbated response by the epithelial cells in addition to resident immune cells may set the stage to augment recruitment and influence the inflammatory response of infiltrating leukocytes when they arrive in the lung. In the context of VN1203, this robust response may overwhelm the host leading to increases in lung tissue damage, decreased respiratory function and overall increases in pathogenesis linked to fatality.

In addition to the known components of the hypercytokinemia response in influenza infection (*IL6, IL29, CXCL10, CCL4*) we have identified several other genes that our model can predict across species, which could be playing roles in initiating and/or sustaining this response. These include components of the NLRP3 inflammasome (*CASP1, NLRP3*, and *PYCARD*) that are coordinately expressed in human cell culture, mouse and macaque in response to VN1203. The inflammasome functions through the action of CASP1, which cleaves the inactive precursor to IL-1β and IL-18, inflammatory cytokines involved in hypercytokinemia. It has been shown to play an essential role in response against influenza virus infection (for a review see [24]), and we have previously shown that it is expressed very early in VN1203 infection in mice [13]. Interestingly, highly pathogenic avian VN1203 viruses are typically lethal in mice, but not uniformly lethal in macaques and humans [12,46]. The trends shown in Figure 2 indicate that the inflammasome may be significantly induced during more lethal infections and might show a more modest response in macaques. The same cluster also contains genes of several transcription factors (*IRF2, IRF5*, and *STAT1*) known to play roles in immune response [47]. STAT1 and the JAK/STAT signaling pathway is known to be targeted by influenza virus [48], but IRF2 and IRF5 have not been demonstrated to play a role in response to influenza infection previously and thus represent novel predictions for further experimental investigation. The inflammasome is known to be an important driver of anthrax toxin-mediated hypercytokinemia [49], and our results here and in a previous study place it in a position to be contributing to VN1203 pathogenesis [13].

Other factors that may be playing a role in hypercytokinemia include factors involved in apoptosis (*XAF1 *and *ATF3*), leukotriene synthesis (*ALOX5*), and lipid metabolism (*CH25H*). We have previously noted that levels of *ALOX5 *were decreased in VN1203 infection in mice, and suggested that this may reflect the increased disease [13], supporting our observations in this study. However, *XAF1 *and *ATF3 *have no reported roles in response to influenza infection and thus represent valuable predictions for further investigation.

Our analysis of common predicted regulatory influences for the most highly cross-predicted genes from our model (Table 4) also provided a number of interesting candidates for further investigation. The most represented predicted regulatory influence, GTF2B, influences over half of the genes. GTF2B, also called TFIIB, is essential for transcriptional preinitiation and guides RNA polymerase II to begin elongation [50]. Interestingly, this general factor is targeted by a variety of viruses to subvert transcriptional initiation for a variety of reasons [51-54]. Additionally, BRF1 is a component of the initiation complex of RNA polymerase III, which is responsible for transcription of ribosomal and other small RNAs, and is known to be modulated by several viruses [55]. Previous reports have shown that influenza virus specifically targets RNA polymerases [56,57], but GTF2B and BRF1 were not specifically identified. Similarly, ATF4 is involved in endoplasmic stress response but is known to be specifically modulated by several viruses [58]. ELF1 and ELF2 are both ETS transcription factors, and FOXE1 is a forkhead family transcription factor involved in thyroid morphogenesis. These factors have no known roles in response to virus, and thus represent novel predictions for further investigation. IFI44 and IFNGR2 are both interferon-related genes and neither is a transcriptional regulator. However, in our model we consider genes that would have indirect effects on transcription as potential influences, and our analysis suggests that these genes may play a role in host response to VN1203. Finally, IRF2 is a known mediator of the interferon response, acting as an attenuator of STAT1 function, and it has been established that influenza virus specifically targets the interferon response through inhibition of the STAT family of activators [48]. However, a specific role for IRF-2 in response to influenza infection has not been established.

## Conclusions

The manipulation of these regulators can be simulated in our model by adjusting their expression levels *in silico*, and simulations used to make specific prediction about the expression of their target genes. Further investigation will be necessary to determine if these factors are important mediators of host response to influenza. These predictions can be first validated in the Calu-3 cell line with minimal effort, for example by examining the consistency of the predictions from perturbed models with experimental results. The results presented here demonstrate that the results should be consistent with what will be observed in the *in vivo *systems, enabling a much quicker and easier experimental validation.

Our findings represent a significant step in the characterization of the relationship between *in vitro *and *in vivo *systems for studying influenza pathogenesis. Additionally, the study presents results showing that portions of the functional response to influenza infection are well-conserved across human cell culture, mouse and macaque in terms of regulatory dynamics. Finally we have identified components of the hypercytokinemia response, which is postulated to cause pathogenesis in VN1203 infection, whose expression can be confidently predicted across species and we have expanded the analysis to implicate several other genes in this important process.

## Methods

### Datasets

We used two transcriptomic data sets for the Calu-3 cells responding to VN1203 influenza infection. The first one has been previously described in [19]. To increase statistical power for the current study, we performed a second Calu-3 experiment, which has a similar design. Calu-3 cells cultured in a 1:1 mixture of Dulbecco's modified Eagle's medium and Ham's F12 nutrient medium (DF12; Invitrogen, Carlsbad, CA) supplemented with 10% fetal bovine serum were washed twice with DF12 containing 0.3% bovine serum albumin (DF12-BSA), and inoculated with influenza A/Vietnam/1203/2004 (VN1203) at a multiplicity of infection (MOI) of 1 plaque forming unit (PFU) per cell, for 50 minutes at 37°C. Following inoculation, monolayers were washed once with DF12-BSA and total cellular RNA was harvested at 0 (immediately after inoculation), 3, 7, 12, 18 and 24 hours post-infection. Duplicate samples were collected from each time point along with time-matched mock-infected controls. RNA was hybridized to Agilent 4X44K human HG (Design ID 014850) arrays and scanned on an Agilent DNA microarray scanner (Model G2505B) using the XDR setting. Extracted raw data were background corrected using the norm-exp method and quantile normalized using Agi4x44PreProcess and RMA Bioconductor packages. Replicate probes were mean-summarized and all probes were required to pass Agilent QC flags for all replicates of at least one infected time point (27,912 probes passed). For modeling the 8471 probes passing QC filtering, a False Discovery Rate (FDR) adjusted p-value < 0.05, and with fold-change expression greater than 1.5 were used.

The transcriptomic data for the macaque study were previously described [12]. Briefly, cynomolgus macaques (*Macaca fascicularis*) were infected with the VN1203, 1918HA/NA:A/Texas/36/91 (1918HANA), 1918HA/NA/NS:A/Texas/36/91 (1918HANAS), and the H1N1 A/Texas/36/91 (Texas91) viruses and response was assessed in lung tissue from two animals on days 1, 2, 4, and 7 postinfection by microarray. Relative expression was assessed by comparing results from infected macaques with pooled samples from seven uninfected macaques.

Twenty-week-old C57BL/6 mice were infected by intranasal instillation of 10^3^, 10^4 ^or 10^5 ^PFU of VN1203 in 50 μl of PBS or mock-infected with PBS alone. At days 1, 2, 4 and 7 days post-infection, lungs were harvested and total RNA was isolated as previously described [19]. RNA was hybridized to Agilent mouse GE 4x44K v2 microarrays. Data acquisition, quality control and differential expression analysis were identical to the Calu-3 experiments described [19]. In the current study we excluded probes that had p-values greater than 0.05 or a maximum fold-change of less than 2.0, leaving a total of 3026 probes for analysis including 523 homologs that were also differentially expressed in the Calu-3 dataset (Additional File 5).

Microarray data described in this study has been deposited in the GEO database under accession numbers GSE33142 and GSE28166 (Calu-3 data), GSE33263 (mouse data), and GSE33351 (macaque data).

### Ethics Statement

All mice were used at 20 weeks of age according to the protocol approved by the University of Wisconsin School of Veterinary Medicine Institutional Animal Care and Use Committee (IACUC). The animal committee mandates that institutions and individuals using animals for research, teaching, and/or testing must acknowledge and accept both legal and ethical responsibility for the animals under their care, as specified in the Animal Welfare Act (AWA) and associated Animal Welfare Regulations (AWRs) and Public Health Service (PHS) Policy. Animal experimentation was done as per the PHS Policy on Humane Care and Use of Laboratory Animals as described in the Guide for the Care and Use of Laboratory Animals.

### Functional enrichment analysis

We used a biological function-centric approach to compare the responses of different experimental models to influenza infection. Three distinct studies were used in the functional comparison; Calu3-cells exposed to VN1203 avian influenza and macaques infected with either VN1203 or a seasonal H1N1variant, with multiple time points in each study. In all, there were 22 distinct treatments (host; virus; time; biological replicate) being compared. For each treatment we determined a list of differentially expressed genes, which were both statistically significant and had a greater than 2-fold change compared to control. We compared each differentially expressed gene list with the list of all the genes in the respective microarrays to determine the functional enrichment p-values for all biological process terms in the gene ontology (GO). Requiring that a GO-term have a p-value < 10^-4 ^in at least one of the treatments resulted in a set of 211 biological process terms that were spread across the various levels of the ontology, and thus vastly differed in the amount of information that they conveyed. In order to remove terms that were too broad or too specific from this list, we quantified the information content for each GO term as *IC_n _*= -log_2_(*N*_*n*_/*N*_*tot*-_) where *IC_n _*is the information content of the *n*^th ^GO term, *N_n _*is the number of genes annotated with this term, and *N_tot _*is the total number of genes with biological process term annotations [21]. We computed IC values for the 211 GO terms using the human genome annotations, and obtained a list of 76 moderately-specific terms with *IC *in the range from 5 to 7.5. We constructed a 76 × 22 enrichment score [-log_10_(*p*) ] matrix, ***E ***to quantify the enrichment of the various process terms (rows) across the various treatments (columns). We then used the dimensionality reduction approach described below to obtain a concise picture of the similarities and differences between the treatments with respect to the biological processes that they affect.

We first clustered the columns of ***E ***to obtain treatment groups that resulted in similar process enrichment profiles. We employed hierarchical clustering with Ward's method of average linkage [59] and clustered the treatments into 5 distinct groups based on the dendrogram. We note that Ward's method uses the increase in the within-cluster sum of squares in evaluating whether two clusters should be joined, and consequently leads to the smallest increase in k-means cost during clustering. Performing k-means clustering of the treatments to obtain 5 clusters results in the exact same treatment grouping as obtained through hierarchical clustering with Ward's linkage. We projected the enrichment scores for the various treatments onto the coordinates for the top three principal components (reduced representation of the 76-dimensional biological process space) to visually evaluate the treatment clustering.

In order to further reduce the dimensionality of the enrichment score matrix we clustered the GO terms (rows of ***E***) based on semantic similarity using the method described by Speer, *et al. *[22]. Briefly we computed a 76 × 76 semantic similarity matrix ***S ***with elements given by *S*(*t*_*i*,_*t*_*j*_) = 2*IC*_*A*_/(*IC*_*i *_+ *IC*_*j*_), where *S*(*t*_*i*,_*t*_*j*_) is the similarity between GO terms *t_i _*and *t_j_*; *IC_i_-*, *IC_j _*and *IC_A _*are respectively the information content values of term *i*, term *j*, and the most informative shared ancestor of these two terms on the GO tree. We then applied the following algorithm based on the spectral clustering approach of Ng, *et al. *[60] to group the GO terms:

1. From the *n*×*n *matrix ***S ***and its derived diagonal matrix ***D***, compute the Laplacian matrix ***L ***= ***D***^-1/2 ^***S D***^-1/2^

2. Select a desired number of clusters *K*

3. Find *v*^1^, *v*^2^,.... *v*^K^, the eigenvectors of ***L***, corresponding to the *K *largest eigenvalues

4. Form the matrix ***V_n×k _***= [*v*^1^, *v*^2^,.... *v*^K^] with these eigenvectors as columns

5. Form the matrix ***Y ***from ***V ***by renormalizing each of the rows of ***V ***to have unit norm

6. Use k-means clustering to cluster the n rows of *Y *into *K *clusters by treating each of the rows as points in a *K*-dimensional space

7. Assign the original GO term object *i *to cluster *j *if and only if row *i *of the matrix of the matrix ***Y ***was assigned to cluster *j*.

We repeated the above algorithm for *K *values ranging from 5 to 15 in step 2, and chose the most parsimonious clustering that gave us a Davies-Bouldin index within 10% of the minimum value. The Davies-Bouldin index for a given value of *K *was computed as DB(K)=1K∑i=1kΔ(Ci)+(Cj)δ(Ci,Cj) where Δ (C_i_) represents the sum of distances from the objects within cluster *i *to the centroid of cluster *i*, and δ(C_i_, C_j_) represents the distance between the centroids of cluster *i *and *j*. Using this approach we clustered the 76 process terms into 13 distinct semantically similar groups. For a given process group-treatment cluster combination with *m *processes and *n *treatments we used the geometric mean value of the *m*×*n *individual enrichment scores as a summarizing metric. Overall, hierarchical clustering of treatments based on their biological process signatures, and spectral clustering of GO terms based on semantic similarity enabled us to condense the initial 76 × 22 enrichment score matrix to a 13 × 5 matrix of mean enrichment scores.

### Cross-coexpression analysis

Similar to the previously described differential clustering analysis [61], cross-coexpression analysis first determines the correlation between the expression vectors of all pairs of the **n **genes in one dataset, which can be represented as an **n**x**n **matrix ***G***. This process is then repeated for the second dataset to give matrix ***H ***of the correlation between **m**x**m **gene expression vectors. Both matrices are filtered to leave only homologs between the two organisms and give matrices ***G_1 _***and ***H_1_***. The cross-coexpression matrix ***I ***is given as the mean:

$$I=\left(G_{1}+H_{1}\right)∕2$$

This analysis can easily be extended to more datasets by determining a core set of homologs and taking the mean of all individual coexpression matrices. High values in the matrix ***I ***mean that the pair of genes indicated has high correlation in both datasets examined.

Differences in the number of observations between the two datasets may bias the results, for example in the case that one or both datasets have a small number of observations. To control for this our cross-correlation approach includes a background correction step. The process above is repeated using expression vectors that have been randomly resorted 100 times. This process is used to assign a p-value to a particular correlation value with a detection limit of 0.01.

### Predictive modeling and cross-validation

Our approach to inference of regulatory influence networks using a multivariate regression approach have been reported previously for other systems [27,28]. Our cross-validation approach used here (Figure 3) was very similar. In the following we refer to the set of target clusters, inferred regulatory influences, and the equations that describe the relationships between their expression levels as a 'predictive model'. Filtered transcriptomic data was clustered using hierarchical clustering (Euclidean distance, complete linkage) and divided into clusters based on the resulting dendrogram. The resulting clusters were used as targets for inference of regulatory influences using the Inferelator [25,26], which uses multivariate regression with the L_1 _penalty, least angle regression. This approach infers a parsimonious set of potential regulatory influences that provide the best description of the mean gene expression, over time and/or under different conditions, of each target as an ordinary differential equation (ODE).

The final 'trained' model represents the relationship between the expression level of a target (y) and the expression levels of regulators with influences on y (X) as an ODE with the form:

(1) $$\tau \frac{dy}{dt}=-y+\sum \beta_{j}X_{j}$$

Here, *τ *is the time step used in model construction and ß is the weight for relationship × on y as determined by L_1 _shrinkage using least angle regression [62]. To predict the expression level of a target cluster eq. 1 can be solved for y. Assuming equilibrium conditions the derivative dy/dt is 0 and so equation (1) can be represented simply as a linear weighted sum:

(2) $$y=\sum \beta_{j}X_{j}$$

In this study we have used an assumption of equilibrium conditions. This assumption then treats each observation as independent from the others (for example, proximal time steps), but simplifies calculations and does not result in a loss of performance for this application (data not shown). For determination of regulatory influences we considered only regulators with expression patterns that were correlated with the target at levels below 0.9. This threshold was used to reduce the number of predicted regulatory influences that are based on correlation, but are not true causal influences.

To ensure that the learned model is able to generalize to novel conditions, we used a cross-validation approach in which each set of related conditions, or individual time points, are removed from the data used for inference. A model inferred from the data set lacking this group of observations is then used to predict the expression of target clusters by applying the model to the held-out data. The performance of the model on that cluster is assessed as the correlation between the predicted expression profile over all conditions versus the observed expression. An overall performance measure for the entire model is obtained as the mean of the performance for each target normalized for the number of genes in that target, giving a mean correlation per gene.

### Cross-predictive application of inferred models across species

The predictive models learned from the Calu-3 expression data were applied to the macaque transcriptional data in a process we refer to as 'cross-prediction'. The predicted expression level for each target cluster, defined by clustering the Calu-3 data as described in the macaque experiment is given by combining the expression levels of the inferred regulators in the macaque experiment with the weights of its influence relationship as inferred from the Calu-3 data (see Figure 3, point 4). This can be expressed as:

$$Y_{Mi}=\overset{k}{\underset{i=1}{\sum }}X_{MA}W_{CAi}+X_{MB}W_{CBi}+X_{MN}W_{Ni}$$

where Y is the predicted expression in macaque (M) of target *i*, K is the number of target clusters considered, and W is the weight of the inferred relationship of inferred regulatory influences (A,B,N) on target i, derived from Calu-3 data (C). The performance of the model is assessed as for cross-validation, as the correlation of the predicted and observed expression profiles over all conditions in the macaque data. Overall performance is calculated as the mean correlation of predicted and observed expression profiles over all conditions, in all target clusters, normalized to the number of genes.

We attempted to better define true regulatory modules that were cross-predictive. To do this we constructed models using between 10 and 120 clusters. For each gene in this analysis we then identified the cluster (from 1495 clusters total) that gives the maximum Xpred score.

We define the Xpred score as a combination of two measures. The first is the correlation of the predicted and observed expression profiles in a given target cluster (P). The second is the correlation of the expression profile from the individual gene with the predicted expression for that target cluster (E). The Xpred score is calculated as:

$$\text{Xpred}\;\text{score}=\begin{array}{cc} \text{PE} & :\text{if}\;\text{P}\;\text{and}\;\text{E}>0\\ -\text{PE} & :\text{if}\;\text{P}\;||\;\text{E}<0 \end{array}$$

This measure provides a way of ranking the predictions based on the performance of the target, and also on the behavior of the individual gene in that target. We show plots of the distribution of these three measures (P, E, and Xpred score) from all genes in Additional File 10.

To assess the significance of the Xpred score we performed 25 randomizations of the genes in each target cluster, then calculated a Zscore for the real prediction as the number of standard deviations from the mean of the randomized scores.

### Canonical Pathway Analysis

Analysis of canonical pathways was performed with Ingenuity Pathways Analysis (Ingenuity Systems). This software analyzes molecular data in the context of known biological response and regulatory networks as well as other higher-order response pathways. Ingenuity functional analysis identified biological functions and/or diseases that were most significant enriched and generated *p*-value to determine the probability that each biological function assigned to that data set was due to chance alone. Enrichment p-values of <0.05 were considered statistically significant. In the functional networks, genes are represented as nodes, and the biological relationship between two nodes is represented as an edge (line). All edges are supported by at least one published reference or from canonical information stored in the Ingenuity Pathways Knowledge Base.

## Authors' contributions

JEM conceived of and designed the study, wrote the algorithms, performed the analyses and drafted the manuscript. HS wrote the functional analysis algorithms and performed the analysis. AJE and GN carried out the cellular infection experiments and provided biological direction and oversight. SEB performed functional analyses and contributed to biological direction. CL performed the mouse infection experiments. SM processed the transcriptional data. CS, YK, MGK and KMW conceived of the study, and participated in its design and coordination and helped to draft the manuscript. All authors read and approved the final manuscript.

## Supplementary Material

Additional file 1 **Supplemental information**; Supplemental methods and results for the manuscript. Click here for file

Additional file 2 **Table S1**; Cross-coexpression analysis of mouse, macaque and human Calu-3 cell response to influenza infection. Click here for file

Additional file 3 **Table S2**; Supplemental table showing transcripts with conserved dynamics in Calu-3 cells, mouse and macaque responding to VN1203 infection. Click here for file

Additional file 4 **Table S3**; Supplemental table showing cluster membership of the 10 cluster model with macaque information. Click here for file

Additional file 5 **Table S4**; Numbers of genes and mouse or macaque homologs differentially expressed in each cluster. Click here for file

Additional file 6 **Hierarchical prediction of macaque expression using Calu-3 model**. The Calu-3 expression data was clustered into different numbers of clusters (X axis) and used to infer models that were used to cross-predict expression in macaque. Individual genes are shown as rows and their performance in cross-prediction is indicated by color, from blue, -1.0, to yellow, 1.0 correlation between predicted and observed expression in macaques. Click here for file

Additional file 7 **Table S5; Top biological functions (as determined by IPA)**. Most statistically significant functions for set were determined and then sorted by # of genes within function. Redundant biological functions (with redundant gene content) were removed and table was limited to 10 biological functions. Click here for file

Additional file 8 **Known relationships between highly predicted genes**. This network depicts all of the molecules within the gene set that were directly or indirectly related using information in the IPA knowledge base. Molecules shaded in grey are represented within the gene set that could be highly predicted in macaques by the Calu-3-based model. This illustrates the interactions between upstream regulators response (ATF3, FOS, JUN), several cytokines and chemokines, interferon-regulated molecules. Click here for file

Additional file 9 **Predicted versus observed expression profiles for IL-6 in mouse**. Click here for file

Additional file 10 **Distribution of prediction scores**. **A. Histogram of cluster prediction correlations**. The correlation of predicted to observed expression profiles was calculated for each cluster considered and is plotted as a histogram, where the frequency indicates the number of genes with that correlation. **B. Histogram of gene correlation with predicted expression**. The correlation of individual gene expression profiles with the predicted expression profile for the cluster that the gene is a member was calculated and is plotted as a histogram. **C. Histogram of the cross-prediction scores**. Cross-prediction scores (cluster prediction × gene correlation) were calculated for all genes in all clusters and are plotted as a histogram. The top × axis indicates the Z scores. Click here for file
