# Supplementary material for: Conserved host response to highly pathogenic avian influenza virus infection in human cell culture, mouse and macaque model systems
Source: BMC Syst Biol. 2011 Nov 11;5:190. doi: 10.1186/1752-0509-5-190 (PMC3229612; doi:10.1186/1752-0509-5-190)
Supplement: Additional file 1 — Supplemental information; Supplemental methods and results for the manuscript. [file 1752-0509-5-190-S1.DOCX]

**Supplemental Methods and Results**

***Evaluation of cross-coexpression analysis approach***

To examine the difference between standard correlation and our cross-coexpression method, we performed three different comparisons. In the first comparison we used data from the two experiments with matched time points, mouse and macaque. We focused only on response to VN1203 infection in macaque and a single dosage in mouse and averaged across biological replicates to obtain single values for each time point. We then calculated correlation between homologs in each set. This comparison represents what would normally be done to compare expression across systems. In the second comparison we used the same two datasets, but used our cross-coexpression approach. We calculated the pairwise correlation between all transcripts with homologs in the macaque dataset, this process was then repeated in the mouse dataset to yield two identically sized matrices. Matrices were combined by averaging corresponding correlation values. The result of this analysis is a score that is the average correlation of a pair of genes in each dataset. High scores indicate that the two genes have similar behavior in both datasets, though their dynamics may be different in each. In the final comparison we repeated the cross-coexpression analysis using an expanded macaque dataset with all viral strains and biological replicates and an expanded mouse dataset with all dosages and biological replicates. This makes the comparison more stringent by requiring cross-coexpressed gene pairs to correlate over a greater number of conditions.

First we compared the ability of the standard approach (referred to here as inter-correlation) to identify genes with similar dynamics between the two datasets with matched time points, mouse infected with 10^5 plaque forming units with biological replicates averaged and macaque infected with VN1203 with biological replicates averaged. We identified 585 transcripts for which homologs were differentially expressed in both datasets. We then examined the inter-correlation between the matching transcripts in both mouse and macaque. This analysis revealed that only about 9% of transcripts (52 of 585) had highly conserved (R >= 0.9; p-value < 0.03) dynamics across both systems. These homologs exhibit very similar temporal dynamics in both mouse and macaque infections.

Cross-coexpression analysis of the same dataset revealed 6% of transcript pairs exhibiting mean correlation values greater than 0.9 (p-value < 0.01), but these relationships covered 98% of the transcripts (574 of 585). This indicates that many pairs of transcripts are expressed in similar patterns in mouse and also in macaque infection.

Because our comparisons are limited to transcripts with homologs in both organisms there are a limited number of data points being compared and this number probably contains some false positive correlations. To examine this possibility, we considered all conditions from both datasets, data from four viral strains with two biological replicates in macaque and data from different viral dose and four biological replicates in mouse. This revealed that 2% of the relationships had a high mean correlation and these relationships covered 64% of the transcripts considered.

This analysis is summarized in Additional File 2 and shows that cross-coexpression analysis is able to identify groups of genes that have similar trends in both organisms, even when those trends are not the same across organisms. This is important information when comparing responses in two (or more) organisms because it allows assessment of similarity of response even when the temporal dynamics are different and when the time courses used do not match each other. Additionally, it allows incorporation of arbitrary numbers of different datasets, for example multiple biological replicates for each dataset.

To identify gene clusters with correlated behavior in all three systems we extended our cross-coexpression analysis to include the Calu-3 cell culture data. This was accomplished by taking the mean of the pairwise gene correlation from each of the individual datasets for those genes with homologs in all three organisms. This analysis resulted in 1% of the relationships with high correlation (311 of 23653) representing 45% of the homologs (98 of 218). While this indicates that the addition of data from another organism decreases the number of genes with high correlation, it is still possible to identify a large number of genes with conserved co-expression across all three organisms. Groups of genes acting as a group in all three organisms during response to influenza infection increases confidence that the response of the group is conserved, even if the trends in the individual organisms are different. In this case the conservation is likely to be at the level of the driving regulatory influences and their relationships with the target genes in the group.

***Construction of fine-grained predictive models using Calu-3 transcriptomics***

Hierarchical clustering will provide divisions of the data into co-expressed clusters with varying degrees of similarity, but it is unlikely that one clustering height (signifying the similarity within the cluster) will provide the best clusters for all cases. We therefore devised an approach to determine the inferred regulatory influences that provide the maximal predictive value from a large number of clusters of various sizes. We used the transcriptomic data from the Calu-3 experiment to generate a hierarchical clustering dendrogram. We then built a series of models using our cross-validation approach with varying numbers of target co-expressed clusters, from 5 to 120. Predictive models were constructed for each set of targets as described above using the Calu-3 and macaque data to infer separate models. Performance of the model was assessed for each co-expressed cluster in each model (1500 clusters total) on the dataset not used in its construction. The performance of the model for each gene was then assessed using a score that combines the correlation of the predicted and observed expression for the gene’s parent cluster in each model and the correlation of the expression profile of the individual gene with the predicted expression profile for the cluster. This score (see Methods) provides a reasonable way of discriminating genes that have profiles that can be predicted well by the model. The results of this analysis are presented as a heatmap in Additional File 8 showing the distribution of the Xpred score over genes in the models. In Additional File 8 each gene (rows) at each dendrogram sampling height (columns) is represented as a heatmap, with high Xpred score (i.e. good prediction) as yellow and negative Xpred score (i.e. poor prediction) as blue. As a control we performed 25 randomizations of the macaque genes with respect to their cluster membership, then assessed the Z score of the Xpred score relative to the randomized background.
