# Supplementary material for: Conserved host response to highly pathogenic avian influenza virus infection in human cell culture, mouse and macaque model systems
Source: BMC Syst Biol. 2011 Nov 11;5:190. doi: 10.1186/1752-0509-5-190 (PMC3229612; doi:10.1186/1752-0509-5-190)
Supplement: Additional file 2 — Table S1; Cross-coexpression analysis of mouse, macaque and human Calu-3 cell response to influenza infection. [file 1752-0509-5-190-S2.DOCX]

**Table S1. Cross-coexpression analysis of mouse, macaque and human Calu-3 cell response to influenza infection.**

|  | **Highly correlated (R>0.9)** | | | | **Overall** | |
| --- | --- | --- | --- | --- | --- | --- |
| **Analysis method** | **%Relationships** | **%Genes** | **#Relationships** | **#Genes** | **#Relationships** | **#Genes** |
| Matched inter-correlation | *NA* | 9% | *NA* | 52 | *NA* | 585 |
| Cross-coexpression (matched) | 6.4% | 98% | 10957 | 574 | 170820 | 585 |
| Cross-coexpression (all) | 2.4% | 62% | 4074 | 365 | 170820 | 585 |
| Cross-coexpression (Calu-3) | 1.3% | 45% | 311 | 98 | 23653 | 218 |
