# Supplementary material for: Conserved host response to highly pathogenic avian influenza virus infection in human cell culture, mouse and macaque model systems
Source: BMC Syst Biol. 2011 Nov 11;5:190. doi: 10.1186/1752-0509-5-190 (PMC3229612; doi:10.1186/1752-0509-5-190)
Supplement: Additional file 5 — Table S4; Numbers of genes and mouse or macaque homologs differentially expressed in each cluster. [file 1752-0509-5-190-S5.DOCX]

**Table S4. Numbers of genes and mouse or macaque homologs differentially expressed in each cluster.**

|  | **Number of genes/homologs** | | |
| --- | --- | --- | --- |
| **Cluster** | **Calu-3** | **Macaque** | **Mouse** |
| **1** | 245 | 174 | 20 |
| **2** | 77 | 47 | 8 |
| **3** | 350 | 159 | 33 |
| **4** | 687 | 277 | 41 |
| **5** | 517 | 344 | 37 |
| **6** | 3067 | 2118 | 182 |
| **7** | 5 | 3 | 1 |
| **8** | 982 | 370 | 62 |
| **9** | 965 | 688 | 65 |
| **10** | 1576 | 724 | 74 |
