# Supplementary figures and images for: Conserved host response to highly pathogenic avian influenza virus infection in human cell culture, mouse and macaque model systems
Source: BMC Syst Biol. 2011 Nov 11;5:190. doi: 10.1186/1752-0509-5-190 (PMC3229612; doi:10.1186/1752-0509-5-190)

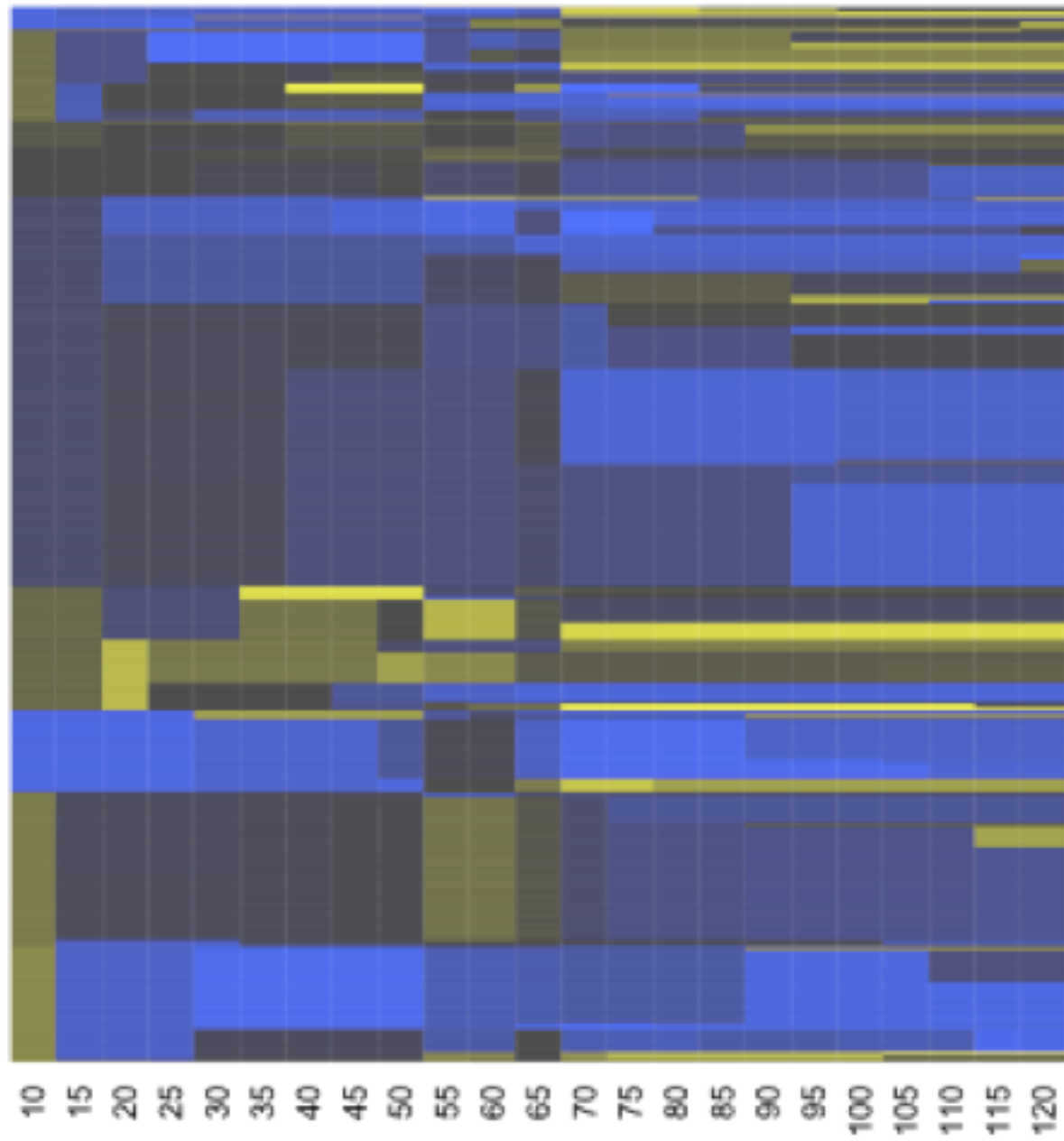

Supplement: Additional file 6 — Hierarchical prediction of macaque expression using Calu-3 model. The Calu-3 expression data was clustered into different numbers of clusters (X axis) and used to infer models that were used to cross-predict expression in macaque. Individual genes are shown as rows and their performance in cross-prediction is indicated by color, from blue, -1.0, to yellow, 1.0 correlation between predicted and observed expression in macaques. [file 1752-0509-5-190-S6.PDF]

Extracellular Space

Plasma Membrane

Cytoplasm

Nucleus

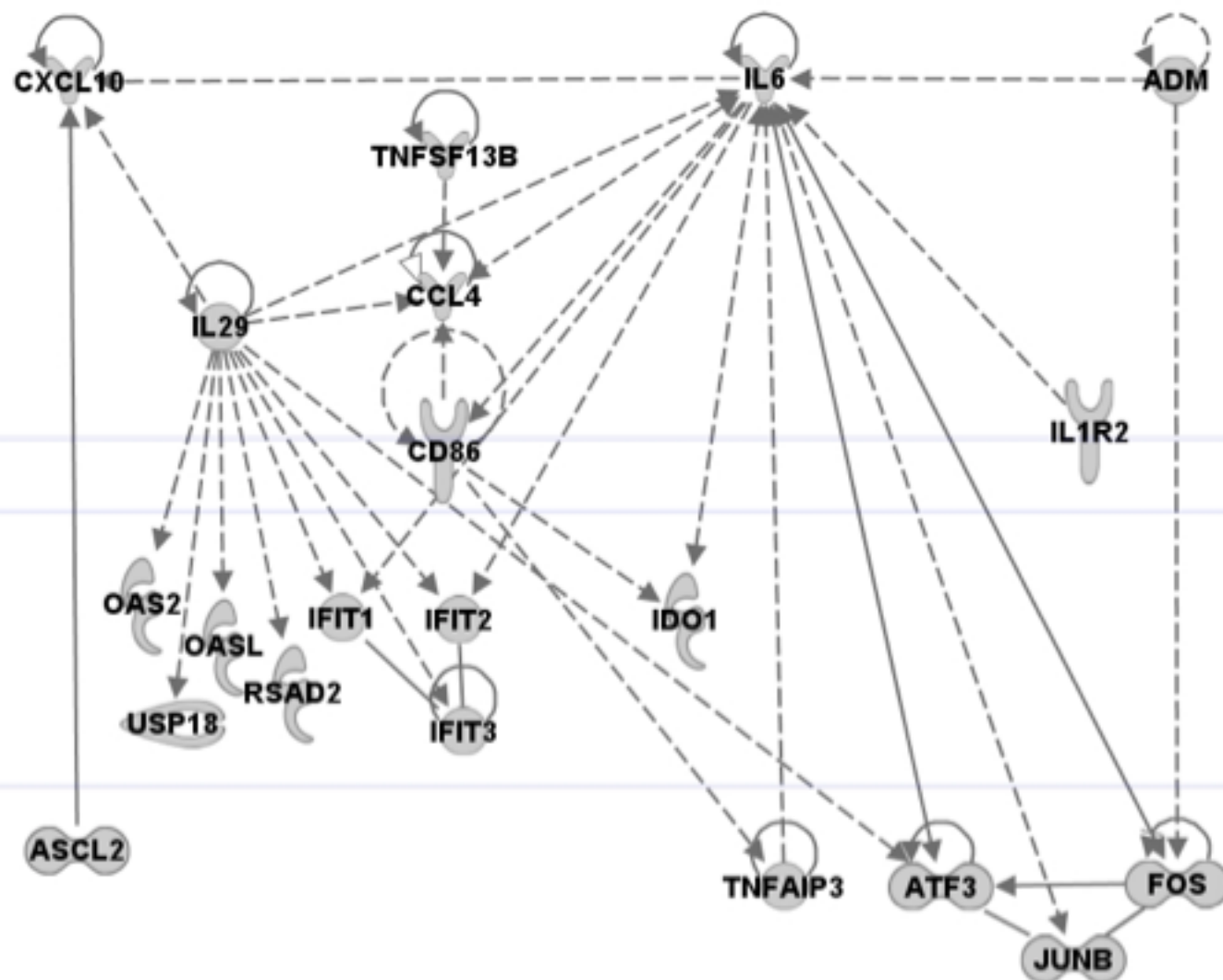

Supplement: Additional file 8 — Known relationships between highly predicted genes. This network depicts all of the molecules within the gene set that were directly or indirectly related using information in the IPA knowledge base. Molecules shaded in grey are represented within the gene set that could be highly predicted in macaques by the Calu-3-based model. This illustrates the interactions between upstream regulators response (ATF3, FOS, JUN), several cytokines and chemokines, interferon-regulated molecules. [file 1752-0509-5-190-S8.PDF]

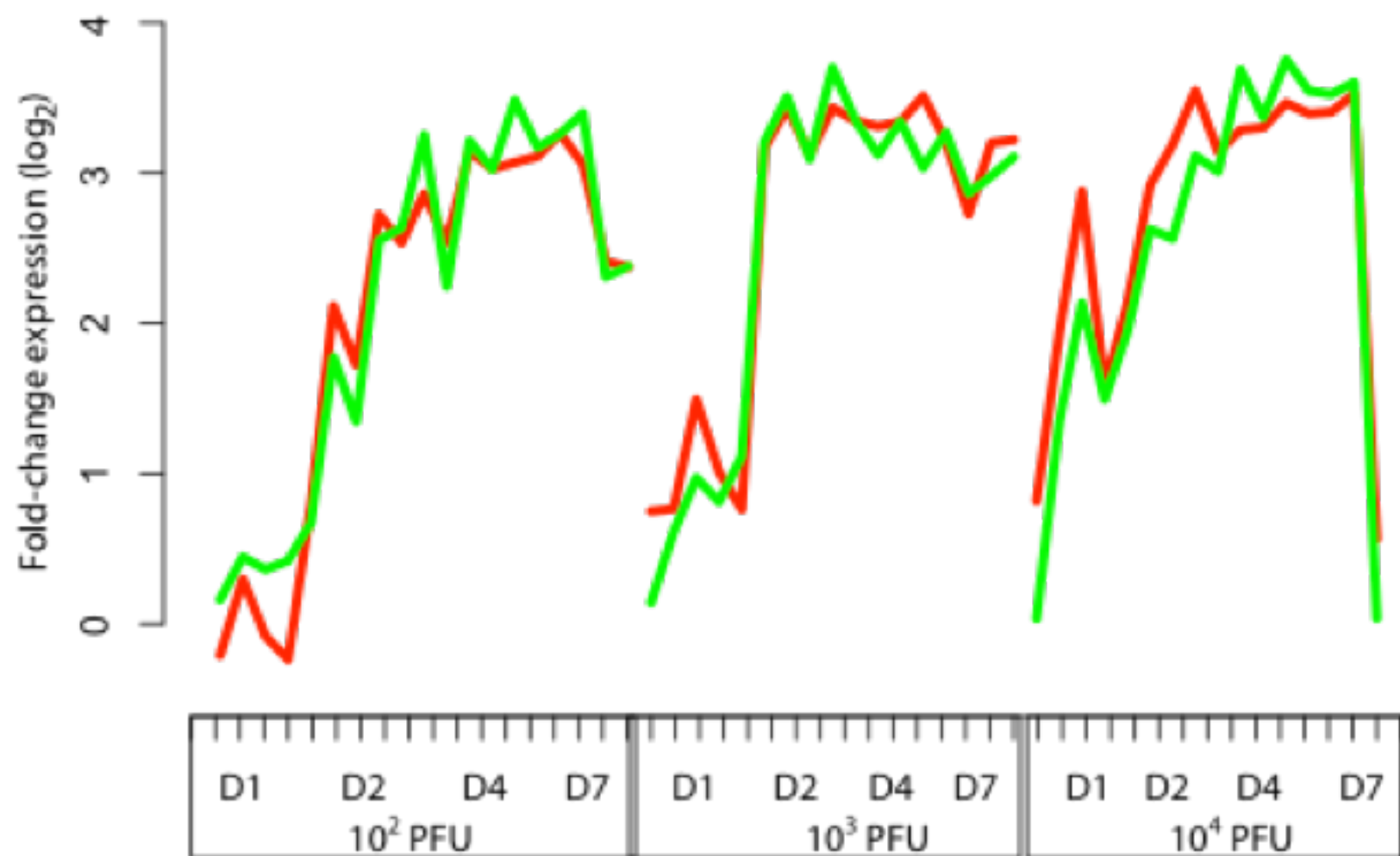

Supplement: Additional file 9 — Predicted versus observed expression profiles for IL-6 in mouse. [file 1752-0509-5-190-S9.PDF]

**A**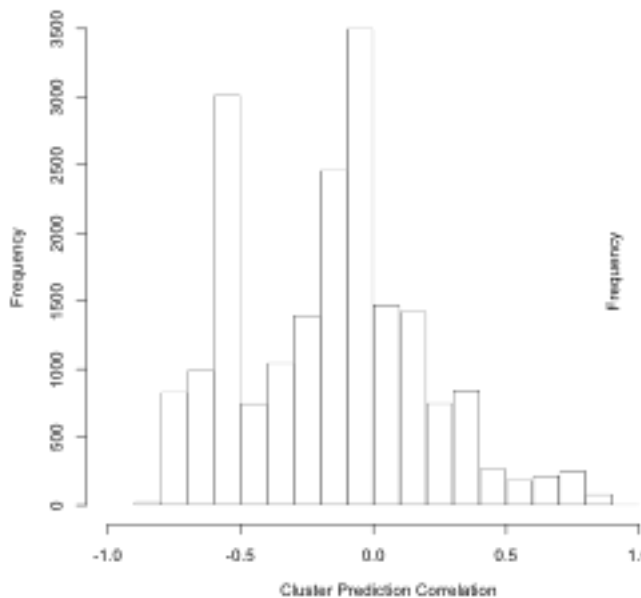**B**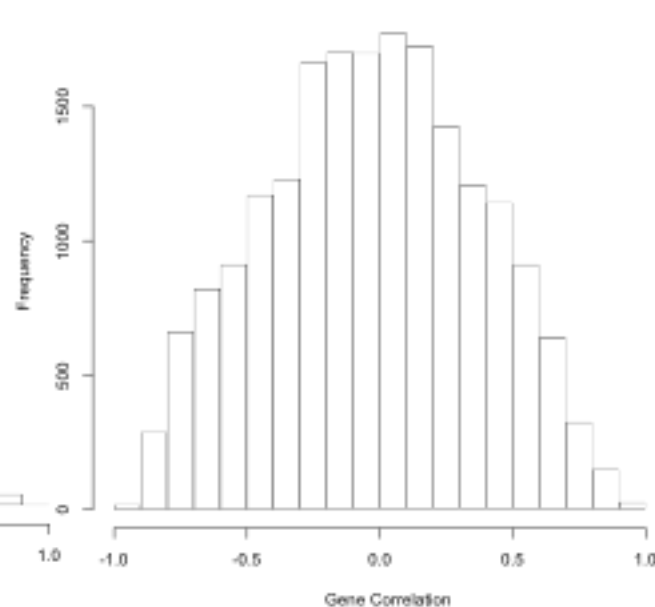**C**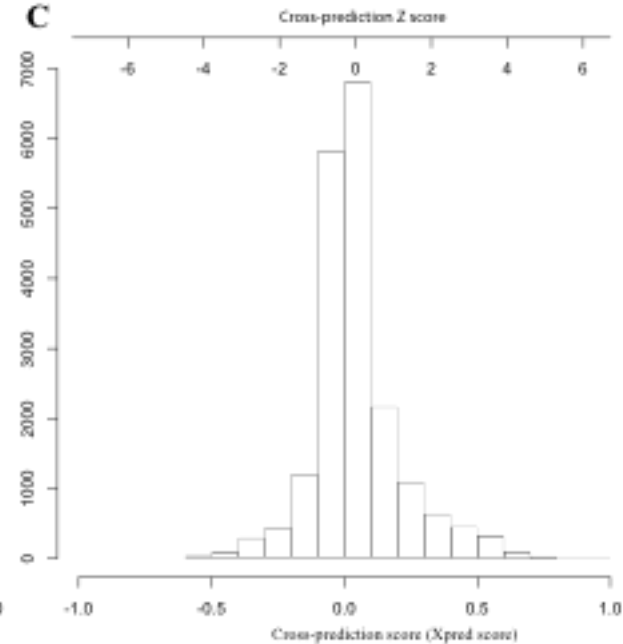

Supplement: Additional file 10 — Distribution of prediction scores. A. Histogram of cluster prediction correlations. The correlation of predicted to observed expression profiles was calculated for each cluster considered and is plotted as a histogram, where the frequency indicates the number of genes with that correlation. B. Histogram of gene correlation with predicted expression. The correlation of individual gene expression profiles with the predicted expression profile for the cluster that the gene is a member was calculated and is plotted as a histogram. C. Histogram of the cross-prediction scores. Cross-prediction scores (cluster prediction × gene correlation) were calculated for all genes in all clusters and are plotted as a histogram. The top × axis indicates the Z scores. [file 1752-0509-5-190-S10.PDF]
